# Discovery of plastic-degrading microbial strains isolated from the alpine and Arctic terrestrial plastisphere

**DOI:** 10.3389/fmicb.2023.1178474

**Published:** 2023-05-10

**Authors:** Joel Rüthi, Mattia Cerri, Ivano Brunner, Beat Stierli, Michael Sander, Beat Frey

**Affiliations:** ^1^Swiss Federal Institute for Forest, Snow and Landscape Research WSL, Birmensdorf, Switzerland; ^2^Institute of Biogeochemistry and Pollutant Dynamics, Swiss Federal Institute of Technology ETH, Zurich, Switzerland

**Keywords:** alpine, Arctic, biodegradable plastic, cold-adapted microorganism, microbial strain, plastic degradation

## Abstract

Increasing plastic production and the release of some plastic in to the environment highlight the need for circular plastic economy. Microorganisms have a great potential to enable a more sustainable plastic economy by biodegradation and enzymatic recycling of polymers. Temperature is a crucial parameter affecting biodegradation rates, but so far microbial plastic degradation has mostly been studied at temperatures above 20°C. Here, we isolated 34 cold-adapted microbial strains from the plastisphere using plastics buried in alpine and Arctic soils during laboratory incubations as well as plastics collected directly from Arctic terrestrial environments. We tested their ability to degrade, at 15°C, conventional polyethylene (PE) and the biodegradable plastics polyester-polyurethane (PUR; Impranil^®^); ecovio^®^ and BI-OPL, two commercial plastic films made of polybutylene adipate-co-terephthalate (PBAT) and polylactic acid (PLA); pure PBAT; and pure PLA. Agar clearing tests indicated that 19 strains had the ability to degrade the dispersed PUR. Weight-loss analysis showed degradation of the polyester plastic films ecovio^®^ and BI-OPL by 12 and 5 strains, respectively, whereas no strain was able to break down PE. NMR analysis revealed significant mass reduction of the PBAT and PLA components in the biodegradable plastic films by 8 and 7 strains, respectively. Co-hydrolysis experiments with a polymer-embedded fluorogenic probe revealed the potential of many strains to depolymerize PBAT. *Neodevriesia* and *Lachnellula* strains were able to degrade all the tested biodegradable plastic materials, making these strains especially promising for future applications. Further, the composition of the culturing medium strongly affected the microbial plastic degradation, with different strains having different optimal conditions. In our study we discovered many novel microbial taxa with the ability to break down biodegradable plastic films, dispersed PUR, and PBAT, providing a strong foundation to underline the role of biodegradable polymers in a circular plastic economy.

## 1. Introduction

Plastics are materials containing one or more synthetic polymers and are widely used in the packaging, electrical, automotive, construction and agricultural industries (Plastics Europe, 2021). The annual global plastic production is still rapidly rising, having reached 367 megatons in 2020 (Plastics Europe, 2021). The persistence of conventional plastics in the environment, the excessive usage of single-use plastics and waste mismanagement are causing a significant environmental problem. Plastics endanger aquatic and terrestrial wildlife (Gall and Thompson, 2015; Blettler and Mitchell, 2021) and have adverse effects on soil fauna (Huerta Lwanga et al., 2016) and plant growth (Hartmann et al., 2022). Through direct deposition, oceanic currents, winds and atmospheric transport, plastics reach even the most remote areas on earth, such as pristine alpine and Arctic environments (Allen et al., 2019; Ambrosini et al., 2019; Bergmann et al., 2019, 2022; Brahney et al., 2021).

Reuse and recycling represent the most sustainable solutions in plastic waste management. However, conventional methods, such as mechanical and chemical recycling, have some considerable downsides. Mechanical recycling leads to a loss of polymer quality, which prevents an infinite reuse of the plastic (Gomes et al., 2022), and chemical recycling requires the application of high temperatures and toxic solvents (Thiyagarajan et al., 2022). A promising approach for recycling of hydrolyzable plastics under mild conditions is by depolymerization of polymers using microbial enzymes involved in plastic biodegradation. In order to biodegrade plastic, microorganisms colonize its surface and form a biofilm (Haider et al., 2019). The colonizing microorganisms secrete extracellular enzymes that depolymerize the polymer, yielding shorter chains as well as oligo-, di- and monomers, which are assimilated by microorganisms. The polymer building blocks are then intracellularly metabolized to CO_2_ and H_2_O (Haider et al., 2019), or can be upcycled to valuable products (Lee et al., 2023). Using isolated enzymes instead of living microorganisms allows selective recovery of plastic monomers. These can be used for the production of new plastics, resulting in a circular economy (Thiyagarajan et al., 2022).

The rate of plastic degradation depends on the properties of the material itself (e.g., shape, monomer type, molecular weight, crystallinity, hydrophobicity), as well as abiotic (e.g., temperature, moisture, pH, UV radiation, oxygen availability) and biotic (e.g., microbial biomass and microorganisms present in a particular environment) factors (Tokiwa et al., 2009; Zumstein et al., 2017b; Haider et al., 2019; Mohanan et al., 2020; Zhang et al., 2021). Conventional, non-hydrolyzable plastics like polyethylene (PE) persist in the environment for extremely long periods (Chamas et al., 2020). Biodegradable plastics have been developed to be broken down and utilized by microorganisms. For example, many strains with the ability to degrade polyester-polyurethane (PUR) were identified (Howard, 2002; Brunner et al., 2018). The potential for PUR degradation is often studied using the model dispersion Impranil^®^DLN (Impranil^®^) because it can be used in simple agar clearing assays (Biffinger et al., 2015). Likewise, polylactic acid (PLA) and polybutylene adipate-co-terephthalate (PBAT) have been shown to be rapidly degraded by Actinobacteria isolated from compost at 50 and 55°C, respectively (Kleeberg et al., 1998; Sriyapai et al., 2018). However, some studies have shown that these plastics still need a long time to be mineralized in conditions less favorable for biodegradation (Karamanlioglu et al., 2017; Narancic et al., 2018; Narancic and O’Connor, 2019). In most studies, degradation of plastics has been reported at temperatures of 20°C or higher. However, earth’s terrestrial ecosystems are on average much colder (Lembrechts et al., 2022), and the fate of biodegradable plastics in such environments has been studies less extensively.

Cold-adapted microorganisms produce enzymes that are active at low temperatures (0–30°C; Gerday et al., 2000; Tchigvintsev et al., 2015). Such microbial strains and their enzymes are highly desirable for industrial recycling applications, as they have the potential to save energy and processing costs by omitting heating steps (Cavicchioli et al., 2011). Cold-adapted enzymes often possess a broader substrate specificity compared to their mesophilic counterparts (Bhatia et al., 2021). This trait may be beneficial for depolymerization applications, where polymers of different chain lengths need to be targeted. In addition, the use of cold-adapted enzymes can prevent unwanted side reactions happening at high temperatures, thereby increasing the purity of the product (Cavicchioli et al., 2011). However, the plastic degradation potential of cold-adapted microorganisms has rarely been studied so far. The degradation of biodegradable polymers, mostly polycaprolactone, by microbial strains isolated from deep sea environments has been reported in a few studies (Sekiguchi et al., 2010, 2011). In addition, Urbanek et al. (2017) isolated microbial strains that were able to degrade the polyesters polybutylene succinate-co-adipate, polycaprolactone and polybutylene succinate from soil collected on the Arctic island of Spitsbergen (Urbanek et al., 2017). However, no microbial strains with the ability to degrade other plastics, such as PUR, PLA and PBAT, at temperatures below 20°C have been reported to date. Further, it is not known how nutrient conditions in the environment and under laboratory conditions affect plastic degradation at low temperatures. For example, addition of gelatin to the culturing medium could induce the expression of PLA-degrading enzymes due to the structural similarity of gelatin and PLA (Jarerat and Tokiwa, 2001).

Recently, we have shown that biodegradable plastics based on PLA and PBAT in alpine and Arctic soils select for a microbiome different from that in the surrounding bulk soil (Rüthi et al., 2020). Microorganisms were possibly enriched in this plastic-associated environment, called the plastisphere (Zettler et al., 2013), because of their ability to degrade these plastics, making the plastics a lucrative C and energy source in these oligotrophic soils. In accordance with this, we found an enrichment of genes encoding enzymes potentially involved in polyester degradation, e.g., cutinases, in the alpine soil plastisphere microbiome (Rüthi et al., 2023). Here, our goal was to isolate microbial strains from the plastisphere in several terrestrial cold environments and assess their ability to utilize different plastics as a C and energy source. In an initial screening, we tested the microbial degradation of commercial plastic products at low temperatures. We expected to find microbial strains able to utilize biodegradable but not conventional PE-based plastics. In a second step, we assessed the impact of additional C sources on the degradation of plastics by testing different culturing media. We proposed two possible outcomes: first, culturing media rich in additional C substrates may inhibit plastic degradation because the microbial strains would not invest energy in producing extracellular enzymes to depolymerize plastic when more readily available C is present. Second, the microbial strains might not be able to thrive with the plastic as the sole C source, and other C sources might be needed to enable degradation of the polymer. In addition, the presence of other polymeric substances, such as gelatin, might induce the expression of secreted enzymes catalyzing plastic degradation. In a last step, we sought to verify the hydrolysis of pure PBAT and PLA polymers using an approach based on the quantification of the co-hydrolysis of a fluorogenic probe embedded in the polymer matrix, adapted from Cerri (2022).

## 2. Materials and methods

### 2.1. Plastic types

Information about all the plastic types used in this study is given in Supplementary Table S1. The polyester-polyurethane (PUR) was Impranil^®^DLN-SD, an anionic, alipathic dispersion used, e.g., as textile coating. Ecovio^®^ is a partly bio-based, compostable plastic based on PBAT and PLA and other components not determined not determined in this study. It was purchased at Coop AG (Basel, Switzerland) in the form of compostable waste bags manufactured by Petroplast Vinora AG (Jona, Switzerland). BI-OPL is a plastic based on PBAT, PLA and other components not determined in this study, which was obtained as a commercial mulch film from Oerlemans Plastics BV (Genderen, Netherlands). Low-density polyethylene (PE) was purchased as commercial waste bag manufactured by TopPac AG (Jonschwil, Switzerland). Pure PBAT was provided by BASF SE (Ludwigshafen, Germany) in the form of pellets. Pure PLA polymer was provided by Sulzer Ltd. (Winterthur, Switzerland) in the form of pellets. Three types of PLA with different isomeric ratios according to the manufacturer were used: L-PLA (100% l-lactic acid isomer), D-PLA (100% d-lactic acid isomer) and L/D-PLA (95% l- and 5% d-lactic acid isomers).

### 2.2. Isolation and identification of microbial strains

Microbial strains were isolated from several alpine and Arctic habitats. All strains, except for strain 780 (*Collimonas arenae*), were associated with plastic (plastisphere; Table 1). That strain 780 of *C. arenae* was isolated from an acidic soil containing high concentrations of aluminum in ‘Val Lavirun’ in the Swiss Alps (Wanner et al., 2018). The plastisphere strains were isolated from plastic films buried during laboratory incubations at 15°C in active-layer soil from ‘Muot da Barba Peider,’ Swiss Alps (hereafter labeled “Swiss Alps”), and ‘Villum’, northern Greenland (Rüthi et al., 2020); from plastic buried for 1 year *in situ* in ‘Villum’ (hereafter labeled “Greenland”); and from plastic litter collected on the shores of the Svalbard archipelago during the Swiss Arctic Project 2018 (hereafter labeled Svalbard). Plastic samples were shaken in sterile tap water for 15 min to detach microbial cells from the surface. A 100 μl sample of the resulting solution and 100 μl of serial dilutions up to 1:10^3^ were streaked out on agar plates (media specified in Table 1) with a sterile glass rod. The plates were incubated at 15°C (except for isolate 780 where the temperature was 4°C), which was observed to favor growth of a diverse microbial community, in the dark, and microbial colonies were sampled once a week for 2 months (Adamczyk et al., 2020). Bacterial colonies were spread on new agar plates using sterile toothpicks to obtain single, pure colonies. Fungal colonies were punched out using a sterile hook and transferred to new agar plates. R2A agar was used for all strains for obtaining the single cultures. The single bacterial colonies were used to inoculate 250 μl of liquid R2A cultures in 0.5 ml Eppendorf tubes. After 2 days of incubation 100 μl of 87% glycerol was added to the liquid cultures, which were then frozen in liquid N_2_ and stored at −80°C. Fungal single cultures were transferred to test tubes containing R2A agar and stored at 4°C.

**Table 1:** Microbial strains isolated from Arctic and alpine plastisphere samples.

Strain	Closest related species	NCBI accession number		Sequence similarity	Culturing medium	Environment	Region	Plastic	Plastic origin
		Isolate	Closest related						
B717	*Psychrobacter cryohalolentis*	OQ052996	MH712970.1	100.00%	R2A	Arctic shore	Svalbard	NA	Litter
B725	*Pseudomonas lini*	OQ052997	MK880647.1	100.00%	R2A	Arctic shore	Svalbard	NA	Litter
F737	*Thelebolus globosus*	OQ053174	KM822751.1	100.00%	R2A	Arctic shore	Svalbard	NA	Litter
B749	*Agrococcus baldri*	OQ052998	KF424713.1	99.50%	R2A	Arctic shore	Svalbard	NA	Litter
F755	*Samsoniella hepiali*	OQ053175	MH301320.1	100.00%	R2A	Arctic shore	Svalbard	NA	Litter
B762	*Umezawaea tangerina*	OQ052999	KX502834.1	99.61%	R2A	Arctic shore	Svalbard	NA	Litter
B765	*Kribbella albertanoniae*	OQ053000	KX503027.1	99.25%	R2A	Arctic shore	Svalbard	NA	Litter
B780	*Collimonas arenae*	OQ053001	MT761800.1	99.37%	R2A 1:10	Alpine soil	Val Lavirun	None	None
B796	*Polaromonas glacialis*	OQ053002	MG973003.1	100.00%	R2A	Arctic shore	Svalbard	NA	Litter
F800	*Neodevriesia* sp.	OQ053176	NR_161141.1	93.39%	R2A	Arctic shore	Svalbard	NA	Litter
B896	*Pseudomonas frederiksbergensis*	OQ053003	KR085914.1	100.00%	R2A	Alpine soil	MBP	BI-OPL	Laboratory incubation
B899	*Collimonas pratensis*	OQ053004	CP013236.1	99.40%	R2A	Alpine soil	MBP	BI-OPL	Laboratory incubation
F914	*Linnemannia gamsii*	OQ053177	MH855790.1	100.00%	R2A	Alpine soil	MBP	PE	Laboratory incubation
F918	*Penicillium stoloniferum*	OQ053178	MH865730.1	99.82%	R2A	Alpine soil	MBP	PLA	Laboratory incubation
B920	*Variovorax ginsengisoli*	OQ053005	AB649025.1	100.00%	R2A	Alpine soil	MBP	ecovio^®^	Laboratory incubation
B926	*Streptomyces vinaceus*	OQ053006	KR857378.1	100.00%	R2A	Arctic soil	Greenland	PP	Field incubation
F940	*Mortierella* sp.	OQ053179	MH790889.1	96.31%	MEA	Arctic soil	Greenland	PP	Field incubation
F942	*Pseudogymnoascus pannorum*	OQ053180	MH864914.1	100.00%	R2A	Alpine soil	MBP	PE	Laboratory incubation
F943	*Lachnellula* sp.	OQ053181	MH858769.1	97.18%	R2A	Alpine soil	MBP	PE	Laboratory incubation
B947	*Pseudomonas fluorescens*	OQ053007	KT369945.1	99.64%	R2A	Arctic soil	Greenland	PP	Field incubation
B950	*Streptomyces avidinii*	OQ053008	KR085837.1	100.00%	R2A	Arctic soil	Greenland	PP	Field incubation
B952	*Streptomyces hypolithicus*	OQ053009	JQ422166.1	99.88%	R2A	Arctic soil	Greenland	PP	Field incubation
B957	*Glaciihabitans tibetensis*	OQ053010	MF555712.1	99.75%	R2A	Alpine soil	MBP	BI-OPL	Laboratory incubation
B958	*Rhodococcus sovatensis*	OQ053011	MK726114.1	99.39%	R2A	Alpine soil	MBP	BI-OPL	Laboratory incubation
B964	*Streptomyces* sp.	OQ053012	NR_044431.1	96.76%	R2A	Alpine soil	MBP	ecovio^®^	Laboratory incubation
F966	*Pseudogymnoascus verrucosus*	OQ053182	MF467855.1	100.00%	R2A	Alpine soil	MBP	ecovio^®^	Laboratory incubation
F967	*Pseudogymnoascus roseus*	OQ053183	MK587683.1	100.00%	R2A	Alpine soil	MBP	PE	Laboratory incubation
B985	*Amycolatopsis* sp.	OQ053013	DQ792502.1	96.76%	R2A	Alpine soil	MBP	PE	Laboratory incubation
F1031	*Pseudogymnoascus verrucosus*	OQ053184	MF467855.1	100.00%	R2A	Arctic soil	Greenland	PE	Laboratory incubation
F1034	*Pseudogymnoascus pannorum*	OQ053185	LC085212.1	100.00%	R2A	Arctic soil	Greenland	BI-OPL	Laboratory incubation
B1045	*Methylobacterium brachiatum*	OQ053014	CP033231.1	99.74%	R2A	Arctic soil	Greenland	PE	Laboratory incubation
F1205	*Pseudogymnoascus pannorum*	OQ053186	LC514968.1	100.00%	SELAA	Alpine soil	MBP	BI-OPL	Laboratory incubation
F1207	*Oidiodendron echinulatum*	OQ053187	MH859482.1	99.42%	R2A	Alpine soil	MBP	ecovio^®^	Laboratory incubation
F1261	*Verticillium leptobactrum*	OQ053188	EF641871.1	99.08%	R2A	Alpine soil	MBP	ecovio^®^	Laboratory incubation

Closest related species and NCBI accession numbers were determined using the BLASTn algorithm in the NCBI nucleotide database. The percentage of sequence similarity to the closest related species is given. The culturing medium used for the isolation of each microbial strain is specified. Strain numbers preceded by ‘B’ are bacterial and strain numbers preceded by ‘F’ are fungal strains. NA, not analyzed; SELAA, soil extract plus lactic acid agar (see Supplementary Materials and Methods), MBP, Muot da Barba Peider; PE, polyethylene; PP, polypropylene.

The strains were taxonomically classified by colony-PCR with subsequent sequencing of the 16S rRNA gene for bacteria and the internal transcribed spacer (ITS) region for fungi. Bacterial and fungal colonies were sampled with sterile toothpicks and suspended in sterile Milli-Q water. For fungal strains, three freeze–thaw cycles were performed using liquid N_2_ and a heating block preheated to 95°C in order to break up their cells. The suspensions were then mixed with G2 Hot Start Polymerase (Promega AG, Dübendorf, Switzerland), MgCl_2_, dNTPs, bovine serum albumin (BSA) and primer pairs. For bacterial isolates the primer pairs 27F (5′-AGAGTTTGATCMTGGCTCAG-3′) and 907R (5′-CCGTCAATTCCTTTRAGTTT-3′) were used. For fungi the primers ITS1 (5′-TCCGTAGGTGAACCTGCGG-3′) and ITS4 (5′-TCCTCCGCTTATTGATATGC-3′) were used. PCR products were purified and sequenced by Macrogen Europe (Amsterdam, Netherlands). The obtained sequences were then compared with the National Center for Biotechnology Information (NCBI) nucleotide database, using the BLASTn algorithm to identify the closest related species. Information about all isolated strains is summarized in Table 1. A species cutoff of 98.65% sequence similarity was adopted, as proposed by Kim et al. (2014). Hence, for microbial strains with no database entry closer than 98.65% only the genus name is given. Information about the cultivation media is given in the Supplementary Materials and Methods. Sequences of the isolated strains were uploaded to the NCBI nucleotide database under the accession numbers OQ052996–OQ053014 (bacteria) and OQ053174–OQ053188 (fungi).

### 2.3. Screening for PUR degradation by clear-zone formation

Screening for PUR degradation was performed by placing the microbial strains on mineral medium (MM) agar plates containing Impranil^®^ as sole carbon source (MM + Imp; Supplementary Materials and Methods). For fungi, a round piece of mycelium (1 cm diameter) was punched out and transferred to the center of the opaque MM + Imp plates. Bacteria were transferred and streaked with a sterile inoculation loop. MM + Imp plates containing the strains were incubated at 15°C in the dark and optically evaluated daily for clear zone (‘halo’) formation. Images of the plates were taken at four timepoints (8, 14, 22, and 28 days) and the time of first occurrence, diameter and clearness of the halos were noted.

### 2.4. Screening for degradation of plastic films as sole carbon sources

Initial screening for degradation of plastic films was done using the commercial products ecovio^®^, BI-OPL and PE. BI-OPL mulch film, ecovio^®^ and PE bags were cut into films (*ca.* 4 × 8 cm pieces) and dried at 40°C for 3 days. After drying, the plastic films were weighed (= initial). The films were then sterilized by soaking them in 70% ethanol for 5 min and afterwards dried at room temperature in a sterile fume hood for 12 h. All subsequent steps were carried out under sterile conditions. The dry, sterile plastic pieces were transferred into glass test tubes and 15 ml of mineral medium (MM) was added (Supplementary Materials and Methods). Microbial strains were added by punching out three round pieces (5 mm diameter) of colony/mycelium from densely overgrown agar plates, taking as little agar as possible, and transferring them to the test tubes. Three replicate test tubes were prepared per strain and plastic, and three negative controls containing only sterile MM were included per plastic. The test tubes were thoroughly shaken for 30 s to distribute the microbial cells and covered with a lid inhibiting contamination but allowing gas exchange. The samples were incubated at 15°C in the dark without shaking for 126 days. After the incubation, the plastic pieces were removed from the test tube and sterilized in 70% ethanol. Next, the plastic films were carefully washed with deionized H_2_O to remove residual biomass, but leaving the plastic films intact, and then again dried for 3 days at 40°C before the residual weight was determined.

The weight loss was calculated with the formula:


$$\mathrm{Weight loss}\;\left(\%\right)=\frac{\mathrm{Weight}\;\left(\mathrm{initial}\right)-\mathrm{Weight}\;\left(\mathrm{residual}\right)}{\mathrm{Weight}\;\left(\mathrm{initial}\right)}*100$$

All samples were checked for contaminations by streaking out 1:10^3^ to 1:10^5^ serial dilutions of the culture supernatants on R2A agar and inspecting emerging colonies for their morphology.

### 2.5. Determining the effects of the culturing medium on microbial degradation

The effect of the culturing medium on the degradation of plastics films was tested using the commercial products ecovio^®^ and BI-OPL. BI-OPL mulch film and ecovio^®^ bags were cut into films (*ca.* 6 × 6 cm) and dried at 40°C for 3 days. After drying, the plastic films were weighed (= initial). The films were then sterilized by soaking them in 70% ethanol for 5 min and afterwards dried at room temperature in a sterile fume hood for 12 h. All subsequent steps were carried out under sterile conditions. The dry, sterile plastic pieces were transferred into Petri dishes and a 15 ml aliquot of each tested medium (MM, MM + gel, R2A and R2A + gel; Supplementary Materials and Methods) was added. Petri dishes were used for the incubation because in the initial screening (see section 2.4) microbial growth and degradation in the test tubes often seemed to be limited to the top of the test tube. This could have been due to oxygen limitation in the lower parts of the tubes; Petri dishes were therefore considered more suitable for optimizing the degradation process by allowing the plastic surface to be consistently exposed to oxygen. Microbial strains were added by punching out three round pieces (5 mm diameter) of colony/mycelium from densely overgrown agar plates, taking as little agar as possible, and transferring them to the Petri dishes. Three replicate Petri dishes were prepared per strain, plastic, and growth medium, and three negative controls containing each of the used sterile growth medium only were included per plastic. Microbial strains were chosen based on the results in the initial screening. For ecovio^®^ strains 762 (*Umezawaea tangerina*), 943 (*Lachnellula* sp.), 964 (*Streptomyces* sp.) and 1,205 (*Pseudogymnoascus pannorum*) were tested, and for BI-OPL strains 737 (*Thelebolus globosus*), 800 (*Neodevriesia* sp.), 943 (*Lachnellula* sp.) and 985 (*Amycolatopsis* sp.) were tested. The samples were incubated at 15°C in the dark without shaking for 60 days (instead of 126 days used in the initial screening, see section 2.4). All samples were checked for contamination by streaking out 1:10^3^ to 1:10^5^ serial dilutions of the culture supernatants on R2A agar and inspecting emerging colonies for their morphology.

### 2.6. Polymer quantification

Since the commercial plastic films were multicomponent materials, and depolymerization of the individual polymer components cannot be concluded solely from total weight loss, NMR analysis was performed in order to determine whether the polymer components (PBAT and PLA) of the biodegradable films were degraded by the microbial strains. The mass loss of PBAT, PLA, and other components not further determined in this study, was calculated for all films significantly reduced in total weight in comparison to negative controls in sections 3.3 and 3.4. To this end, residual polymer masses were quantified by ^1^H-NMR as previously reported (Nelson et al., 2019). Solutions of polymers and the internal standard were prepared in deuterated chloroform (CDCl_3_) (DE40EAG, Apollo Scientific) by directly dissolving the films in CDCl_3_. Aliquots (1 ml) of these solutions were transferred into NMR tubes and ^1^H-NMR spectra were collected (Bruker Avance III 400 MHz NMR, Bruker 5 mm BBFO 400 MHz Z Gradient probe; 16 dummy scans and 128 measurement scans with a pulse width of 14 μs and a 15 s delay time between scans). 1,4-dimethoxybenzene (DMB) (D0629, Tokyo Chemical Industry) and 1,4-dinitrobenzene (DNB) (102,369, Sigma-Aldrich) were used as internal standards for the quantification (for the NMR quantification of the degraded samples and the determination of the reference composition of the pristine materials, respectively). The mass of each polymer was calculated from the ^1^H-NMR spectrum according to (Bharti and Roy, 2012):


$$m_{x}=M_{w_{x}}\frac{\#H_{\mathrm{IS}}a_{x}}{\#H_{x}a_{\mathrm{IS}}}n_{\mathrm{IS}}$$

where m_x_ (g) is the mass of the polymer, $M_{w_{x}}$ (g/mol) is the molecular weight of the polymer repeat unit, a_x_ and a_IS_ (arbitrary units) are the areas of the peaks at the characteristic chemical shifts chosen for the polymer and internal standard (Nelson et al., 2019), respectively, #H_IS_ and #H_x_ the number of protons per single molecule/repeat unit contributing to the signal of these peaks and n_IS_ (mol) is the amount of internal standard added to the sample. The mass of other, undetermined components (hereafter called “other”) was calculated by subtraction of the PBAT and PLA masses from the total weight of the plastic films.

^1^H-NMR spectra were processed in MestReNova 14.2.0. Spectra were first referenced to the ^1^H peak of residual non-deuterated CHCl_3_ in the deuterated solvent (7.26 ppm; Fulmer et al., 2010). The phase was manually corrected, and the baseline set to an intensity of approximately zero with a piecewise linear correction, then all characteristic peaks were manually integrated. Spectra of the pristine materials were used as a reference (Supplementary Figure S1 and Supplementary Data S1, S2) to calculate the residual polymer content of the samples after incubation. Pristine ecovio^®^ and BI-OPL contained 64.36 ± 0.89% and 61.12 ± 0.39% PBAT, and 3.02 ± 0.06% and 12.67 ± 0.12% PLA, respectively (*n* = 3). All further data analysis was performed in R 4.2.2 (R Core Team, 2022) and the IDE RStudio 2022.12.0.353 (R Studio Team, 2022).

### 2.7. Screening for enzymatic hydrolysis using a fluorescence-based polymer degradation assay

A fluorescence-based method adapted from Cerri (2022) was applied to quantify hydrolysis of the pure polymers PBAT, L-PLA, D-PLA, and L/D-PLA. The approach is based on a fluorogenic probe embedded in the polymer matrix. Fluorescence is only detected once the fluorogenic probe is co-hydrolyzed together with the polyester polymer.

To prepare 96-well microplates containing the polymer with the fluorogenic probe embedded in it, 150 mg of polymer (PBAT, L-PLA, D-PLA, or L/D-PLA) was dissolved in a solvent mix containing 9 ml CHCl_3_ and 1 ml C_2_HCl_3_ by sonication. A 20 mM stock solution of 4-methylumbelliferyl laurate (4-MUL; Santa Cruz Biotechnology Inc., Dallas, TX, United States) was prepared in C_2_HCl_3_. A 12.5 μl aliquot of 4-MUL stock solution was added to the dissolved polymer. A 100 μl aliquot of the resulting mixture was pipetted into each well of the 96-well plates (black, flat bottom, polypropylene; Greiner Bio-One VACUETTE Schweiz GmbH, St. Gallen, Switzerland), resulting in a total of 2.5 nm 4-MUL per well. The solvent was then evaporated by placing the plate onto refractory clay pre-warmed to 100°C, resulting in a polymer-coated 96-well plate.

Pre-cultures of microbial strains were prepared at 15°C in 2 ml Eppendorf tubes containing MM and a piece of either ecovio^®^ or BI-OPL film (*ca.* 1 × 2 cm), without shaking. All 34 strains were incubated with ecovio^®^. Further, pre-cultures with BI-OPL films were prepared for all strains that had significantly decreased the weight of BI-OPL in the initial screening. In addition, pre-cultures of strains DSM 44262 (*Amycolatopsis alba*; Leibniz-Institute DSMZ-German Collection of Microorganisms and Cell Cultures) and DSM 50188 (*Pseudomonas oleovorans*, syn. *Pseudoalcaligenes*; Leibniz-Institute DSMZ-German Collection of Microorganisms and Cell Cultures) were prepared with ecovio^®^ in MM and incubated at 28°C as positive controls. Strain DSM 44262 (*Amycolatopsis alba*) was chosen as a PLA-degrading positive control (Butbunchu and Pathom-Aree, 2019), and strain DSM 50188 (*Pseudomonas oleovorans*) was chosen as a PBAT-degrading positive control (Wallace et al., 2017). Negative controls with MM and plastic film without any microbial strain were included to analyze the background fluorescence. Subsamples (250 μl) were taken and transferred into the polymer-coated plates directly after incubation (t0), 7 days after incubation (t1) and 25 days after incubation (t2) of the pre-cultures.

After the transfer of microbial culture to the 96-well plate, the samples were immediately measured using a plate reader (Tecan Infinite 200 Pro; Tecan Group AG, Männedorf, Switzerland). Excitation and emission wave lengths were set to 325 nm and 450 nm, respectively, and fluorescence was measured every 90 s for 24 h at 30°C. It was not possible to set a lower temperature because the plate reader could not actively cool.

Each plate contained a standard series to calculate the amount of hydrolyzed 4-MUL with a calibration curve. For this step, a 100 μM 4-methylumbelliferone (4-MU; Merck KgaA, Darmstadt, Germany) stock solution was prepared in methanol and diluted with MM to 0 to 11 μM (in 1 μM steps) in the 96-well plates to reach a final volume of 250 μl. The amount of hydrolyzed 4-MU was calculated for each sample using the calibration curve.

As the positive control strains for both PBAT and PLA did not result in an increase in fluorescence, hydrolysis of the polymers by enzymes was additionally measured as a positive control. For PBAT a lipase from *Rhizopus oryzae* (RoL; Merck KgaA, Darmstadt, Germany) was used as a positive control (Zumstein et al., 2017a), whereas for PLA proteinase K from *Tritirachium album* (proK; Merck KgaA, Darmstadt, Germany) was used (Tokiwa et al., 2009). Assays of 4-MUL with *Rhizopus oryzae* lipase and proteinase K were run in MOPS buffer [150 mM gamma-(*N*-Morpholino)propanesulphonic acid, adjusted to pH 7 with KOH] for 9 h in duplicates at 30°C and 37°C, respectively. Assays of 4-MUL with proteinase K were repeated in 50 mM Tris–HCL (pH 8). All enzyme reactions were run with an enzyme concentration of 12 μM and standard series with 4-MU were prepared in the respective buffer. Whereas the assay worked well using PBAT, PLA hydrolysis could not be detected by any microbial strain or by any positive control with the tested PLA materials under the tested conditions. As no working positive control was available, PLA degradation was not further analyzed with this assay.

### 2.8. Statistical analyses

Statistical analyses were carried out using R v4.1.3 (R Core Team, 2022). Plots were generated with the R packages *tidyverse* (Wickham et al., 2019) and *ggpubr* (Kassambara, 2020) and statistical tests were considered significant if *p* < 0.05, unless stated otherwise. The function ‘LeveneTest’ was used to test the homogeneity of variances (package *DescTools*; Signorell, 2021). Differences in total weight loss and mass loss of individual polymer components (PBAT and PLA) of the plastic films were tested with analysis of variance (ANOVA) using the function ‘aov.’ Pairwise comparisons between different strains and the negative controls were done with pairwise t-tests with the function ‘pairwise.t.test,’ and *p*-values were corrected for multiple testing using the false discovery rate ‘fdr.’ To test the effects of the strain, culture medium, and interaction between strain and culture medium on the weight loss in the second part of the study (section 3.4), ANOVA (function ‘aov’) was used, and the function ‘anova’ with the option ‘white.adjust’ = TRUE was used to correct for inhomogeneity of variance for ecovio^®^ samples. *Post hoc* pairwise comparisons with combinations of strains and media were done by pairwise *t*-tests using the function ‘pairwise.t.test,’ and *p*-values were corrected for multiple testing using the false discovery rate ‘fdr.’ To test the hypothesis that Petri dishes favor degradation compared to test tubes, one-sided *t*-tests were performed using the function ‘t.test’ with the options ‘var’ = TRUE and ‘alternative’ = less. Linear correlation between the mass loss and the terephthalate content of the PBAT component of the ecovio^®^ and BI-OPL films was tested using the function ‘stat_cor’ with the method ‘pearson’ (package *ggpubr*; Kassambara, 2020). For the phylogenetic trees, multiple alignments were run using the functions ‘msa’ with the method ‘ClustalOmega’, ‘msaConvert’ (package *msa*; Bodenhofer et al., 2015), ‘dist.alignment’ (package *seqinr*; Charif and Lobry, 2007) and ‘bionj’ (package *ape*; Paradis and Schliep, 2019). For rooting the alignments, sequences from the NCBI nucleotide database from different phyla not isolated in our study were used. For bacteria the 16S sequence of NR_125480.1 *Prochlorococcus marinus* (Cyanobacteria) was chosen, and for fungi the ITS sequence of NR_171237.1 *Cryolevonia schafbergensis* (Basidiomycota) was selected. The phylogenetic trees were plotted using the function ‘ggtree’ (package *ggtree*; Guangchuang, 2020).

## 3. Results

### 3.1. Microbial strains isolated from the plastisphere

In total, 34 microbial strains were isolated from the plastisphere (Table 1). Nineteen strains were obtained from the surfaces of plastic films buried in alpine (16 strains) and Arctic (3 strains) soils during a laboratory incubation experiment (Rüthi et al., 2020). Five strains were isolated from plastic pieces buried in Arctic soil in the field for 1 year. Nine strains were isolated from plastic litter collected on the shores of the Svalbard archipelago and one isolate was obtained from alpine soil not affected by plastic. The 19 bacterial strains belonged to the phyla Actinobacteria (10 strains) and Proteobacteria (9 strains), and the 15 fungal isolates belonged to the phyla Ascomycota (13 strains) and Mucoromycota (2 strains). Phylogenetic trees of the bacterial and fungal strains are shown in Supplementary Figure S2.

### 3.2. Alpine and Arctic microbial strains degrade Impranil^®^ at low temperature

**Figure 1:**
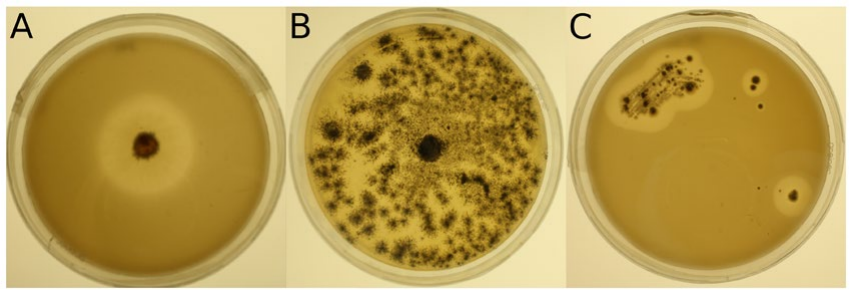
Examples of mineral medium plus Impranil^®^ (MM + Imp) plates inoculated with microbial strains for evaluation of Impranil^®^ degradation. **(A)** Photo of strain 943 (*Lachnellula* sp.) grown on MM + Imp for 7 days. **(B)** Photo of strain 1,205 (*Pseudogymnoascus pannorum*) grown on MM + Imp for 14 days. **(C)** Photo of strain 985 (*Amycolatopsis* sp.) grown on MM + Imp for 28 days. Photos of all tested strains grown on MM + Imp for 28 days are shown in Supplementary Figures S3, S4.

The 34 selected microbial strains were grown on MM + Imp plates to evaluate PUR degradation by formation of cleared zones around the isolates (halos; Figure 1). Of the 34 tested microbial strains, halo formation was observed for 22 strains after 28 days of growth at 15°C (Supplementary Figures S3, S4). However, for strains 950 (*Streptomyces avidinii*), 762 (*Umezawaea tangerina*) and 947 (*Pseudomonas fluorescens*) only very small and barely recognizable halos were observed even after 28 days, leaving their ability to degrade Impranil^®^ disputable. Impranil^®^ degradation was widespread in the presence of fungi (11 out of 15), whereas fewer halos were detected around bacterial strains (8 out of 19, excluding the ambiguous strains mentioned above). Several members of both Actinobacteria and Proteobacteria caused halo formation on MM + Imp plates. The strongest Impranil^®^ degradation by bacteria occurred for the isolates 985 (*Amycolatopsis* sp.), 958 (*Rhodococcus sovatensis*), 717 (*Psychrobacter cryohalolentis*), and 780 (*Collimonas arenae*). For the bacterial strains 717 (*Psychrobacter cryohalolentis*), 780 (*Collimonas arenae*), and 958 (*Rhodococcus sovatensis*), the first signs of halo formation were detected already after 3 days of incubation. Halos around fungi generally were larger and clearer than those around bacteria. For the fungal isolates 737 (*Thelebolus globosus*), 918 (*Penicillium stoloniferum*), 943 (*Lachnellula* sp.), 1,031 (*Pseudogymnoascus verrucosus*), 1,205 (*Pseudogymnoascus pannorum*), and 1,261 (*Verticillium leptobactrum*) the first signs of halo formation were detected already after 3 days of incubation. Interestingly, all *Pseudogymnoascus* strains except one did form a halo on MM + Imp plates. The ability to degrade Impranil^®^ was widespread across the tested Ascomycota, but neither of the two tested Mucoromycota was able to degrade Impranil^®^. For several fungal strains the evaluation of diameter was challenging because the fungi spread over the complete agar plate by producing spores. Images of Impranil^®^ plates for all microbial strains are shown in Supplementary Figures S3, S4.

### 3.3. Alpine and Arctic microbial strains are able to degrade biodegradable plastic films

**Figure 2:**
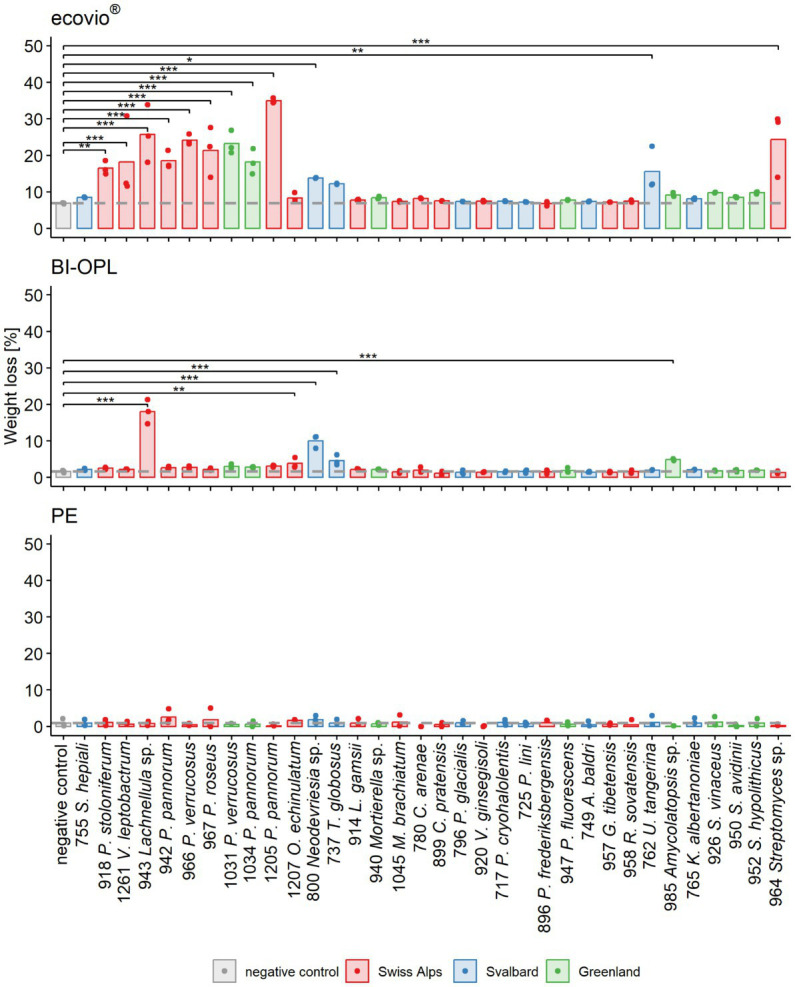
Calculated weight loss determined in the initial screening for each microbial strain and plastic. Weight loss detected for ecovio^®^ (top), BI-OPL (middle), and polyethylene (PE; bottom) films. Dots indicate the weight loss of the samples and bars indicate the means of three replicates. The dashed gray line indicates the mean of the negative controls. Colors indicate the origin of the microbial strains. Asterisks indicate the level of significance between the negative controls and the strains, with ***p < 0.001, **p < 0.01, and *p < 0.05. For strain 755: *S., Samsoniella*; for strain 918: *P.*, *Penicillium, V.*, *Verticillium*; for strains 942, 966, 967, 1,031, 1,034, and 1,205: *P.*, *Pseudogymnoascus*, *O*., *Oidiodendron*, *T*., *Thelebolus*, *L*., *Linnemannia*, *M*., *Methylobacterium*, *C*., *Collimonas*; for strain 796: *P*., *Polaromonas*, *V*., *Variovorax*; for strain 717: *P*., *Psychrobacter*; for strains 947 and 896: *P*., *Pseudomonas*, *A*., *Agrococcus*, *G*., *Glaciihabitans*, *R*., *Rhodococcus*, *U*., *Umezawaea*, *K*., *Kribbella*; for strains 952, 950, and 926: *S*., *Streptomyces*. Detailed information on each microbial strain is given in Table 1.

In an initial screening, all 34 microbial strains were cultured individually at 15°C in MM with no C source (Supplementary Materials and Methods) other than one of the three tested commercial plastics (ecovio^®^, BI-OPL and PE). Most significant weight losses in comparison to negative controls and the largest reductions in total weight were found for ecovio^®^, followed by BI-OPL (Figure 2). No significant weight losses were detected for PE after the 126-day incubation. Of the tested strains, 12 (10 fungi, 2 bacteria) significantly reduced the total weight of the ecovio^®^ and 5 (4 fungi, 1 bacterium) of the BI-OPL films, respectively.

**Figure 3:**
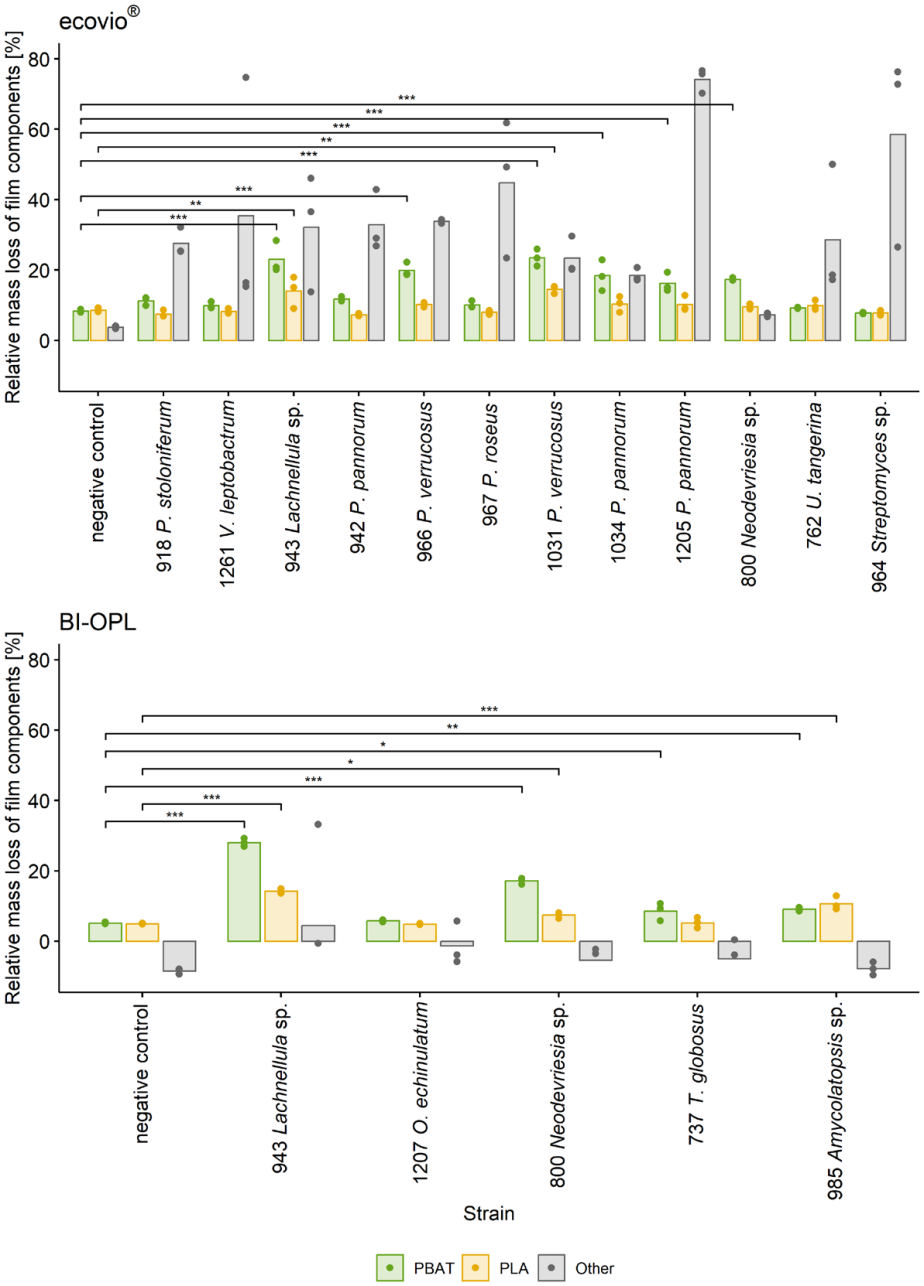
Mass loss of individual components (PBAT, PLA and other) in ecovio^®^(top) and BI-OPL (bottom) films determined by NMR. Only strains with total weight losses significantly different from the negative controls were analyzed. Dots indicate the mass loss of the samples and bars indicate the means of three replicates. Colors indicate the individual film components. Asterisks indicate the level of significance between the negative controls and the strains, with ***P < 0.001, **P < 0.01, and *P < 0.05. No statistical analysis was performed for components other than PBAT and PLA. For strain 918: *P*., *Penicillium*, *V*., *Verticillium*; for strains 942, 966, 967, 1,031, 1,034, and 1,205: *P*., *Pseudogymnoascus*, *U*., *Umezawaea*, *O*., *Oidiodendron*, *T*., *Thelebolus*. Detailed information on each microbial strain is given in Table 1.

All tested Ascomycota, except strains 755 (*Samsoniella hepiali*), 1,207 (*Oidiodendron echinulatum*) and 737 (*Thelebolus globosus*), significantly reduced the weight of the ecovio^®^ films. The largest total weight reductions of ecovio^®^ films were caused by the fungal strains 1,205 (*Pseudogymnoascus pannorum*; 34.9%) and 943 (*Lachnellula* sp.; 25.8%). NMR data showed that strain 943 (*Lachnellula* sp.) significantly degraded the PBAT and PLA fractions of the ecovio^®^ films whereas 1,205 (*Pseudogymnoascus pannorum*) only degraded the PBAT component (Figure 3). For the other *Pseudogymnoascus* strains cultured with ecovio^®^ films significant degradation of the PBAT (966 and 1,031 *Pseudogymnoascus verrucosus*, 1,034 *Pseudogymnoascus pannorum*) and PLA (1,031 *Pseudogymnoascus verrucosus*) components was found. Strain 800 (*Neodevriesia* sp.) significantly degraded the PBAT component of ecovio^®^ films (Figure 3). Actinobacteria strains 762 (*Umezawaea tangerina*) and 964 (*Streptomyces* sp.) significantly reduced the weight of ecovio^®^, but did not significantly degrade the PBAT and PLA, suggesting that only components other than the two polymers were degraded (Figure 3).

For BI-OPL, a significant reduction in total weight occurred with strains 943 (*Lachnellula* sp.; 18.0%), 800 (*Neodevriesia* sp.; 10.0%), 985 (*Amycolatopsis* sp.; 4.9%), 737 (*Thelebolus globosus*; 4.6%), and 1,207 (*Oidiodendron echinulatum*; 3.8%). Strains 943 (*Lachnellula* sp.), 800 (*Neodevriesia* sp.) and 985 (*Amycolatopsis* sp.) significantly degraded the PBAT and PLA in the BI-OPL films (Figure 3). 737 (*Thelebolus globosus*) significantly degraded the PBAT component of BI-OPL films (Figure 3). None of the tested Proteobacteria and Mucoromycota showed significant differences from the negative control for both ecovio^®^ and BI-OPL.

Furthermore, strong linear correlation was detected between the degradation and the terephthalate content of the PBAT component for both ecovio^®^ (*R* = 0.96, *p* = 2.2*10^−16^) and BI-OPL (*R* = 0.97, *p* = 1.0*10^−11^) films (Supplementary Figure S5). Plastic films were visually inspected after the incubation (see images in Supplementary Figure S6). Results of statistical tests are given in Supplementary Tables S2, S3.

### 3.4. The culturing medium strongly affects the degradation of biodegradable plastic films

**Figure 4:**
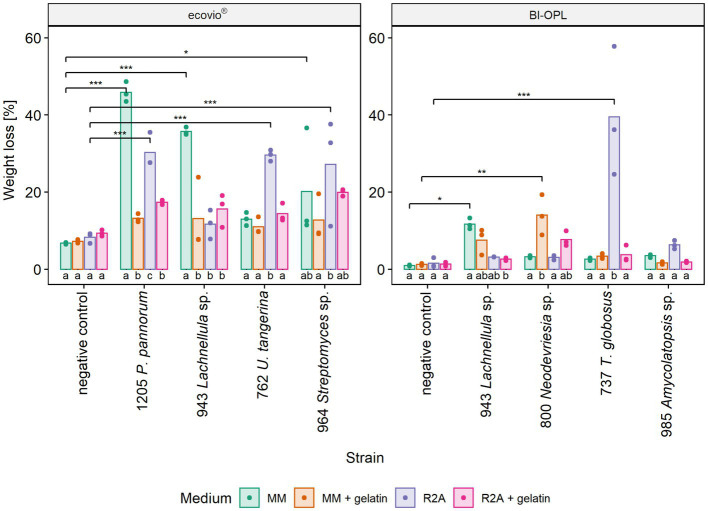
Calculated weight loss of ecovio^®^ and BI-OPL plastic films incubated with selected strains in different culturing media. Dots indicate the weight loss of the samples and bars indicate the means of three replicates. Colors indicate the culturing media. Asterisks indicate the level of significance determined between each strain and the negative control in the corresponding medium, with ***p < 0.001, **p < 0.01, and *p < 0.05. Different letters below the bars indicate significant differences (*p* < 0.05) between the tested media for each strain. *P*., *Pseudogymnoascus*, *U.*, *Umezawaea*, and *T*., *Thelebolus*. Detailed information on each microbial strain is given in Table 1.

The most promising microbial strains, according to total weight loss in the initial screening (section 3.3), were selected for testing the effect of different culturing media on plastic degradation. The impact of the culturing medium was determined by comparing the weight losses of the plastic films after culturing the strains in four different media (MM, MM + gel, R2A, R2A + gel; Supplementary Materials and Methods). For ecovio^®^ strains 943 (*Lachnellula* sp.), 964 (*Streptomyces* sp.) and 1,205 (*Pseudogymnoascus pannorum*) were tested. In addition, strain 762 (*Umezawaea tangerina*) was selected in order to test a second bacterial isolate with ecovio^®^. For BI-OPL strains 737 (*Thelebolus globosus*), 800 (*Neodevriesia* sp.), 943 (*Lachnellula* sp.) and 985 (*Amycolatopsis* sp.) were tested. Results indicate that the culturing medium strongly influenced the degradation of plastic films (Figure 4). However, no clear pattern was found and different strains showed different reactions to the tested media. The effects of the strain and the medium, as well as the interaction between the strain and the medium, were highly significant for both ecovio^®^ and BI-OPL, as shown by ANOVA (Supplementary Table S4). Pairwise comparisons showed no differences between the negative controls in the different media. On the contrary, there were clear differences in the weight losses of the plastic films when the strains were cultured in the different media (Supplementary Table S4). For strain 943 (*Lachnellula* sp.) with both ecovio^®^ and BI-OPL and for strain 1,205 (*Pseudogymnoascus pannorum*) with ecovio^®^, the largest total weight losses were detected when the plastic films were cultured in MM without an additional C source. For both strains both PBAT and PLA in the ecovio^®^ films were significantly degraded when cultured in MM whereas for strain 1,205 (*Pseudogymnoascus pannorum*) both polymers were not significantly affected, suggesting that only other, unknown components were degraded when cultured in R2A (Supplementary Figure S7). Strain 800 (*Neodevriesia* sp.) degraded most BI-OPL in MM + gel followed by R2A + gel (Figure 4). For strains 737 (*Thelebolus globosus*) and 762 (*Umezawaea tangerina*) degradation was most effective in R2A (Figure 4). The same trend was observed for strains 985 (*Amycolatopsis* sp.) and 964 (*Streptomyces* sp.). Interestingly, strain 737 (*Thelebolus globosus*) significantly degraded both PBAT and PLA components of the BI-OPL, and strain 762 (*Umezawaea tangerina*) the PLA component of the ecovio^®^ films when cultured in R2A (Supplementary Figure S7), which was not observed in the initial screening using MM (Figure 3).

Notably, a significantly larger weight loss occurred for ecovio^®^ with strains 943 (*Lachnellula* sp.; *p* = 0.048) and 1,205 (*Pseudogymnoascus pannorum*; *p* = 0.001) in MM when they were cultured in Petri dishes compared with the initial screening in test tubes, even though the incubation period in the Petri dishes was only approximately half as long as that in the test tubes (Supplementary Table S5). Images of the plastic films after the incubation are shown in Supplementary Figure S8 and results of the statistical tests of the mass loss of individual film components are given in Supplementary Table S3.

### 3.5. Fluorescence-based 4-MUL assay indicates the ability of microbial strains to hydrolyze PBAT

The ability of the microbial strains to hydrolyze PBAT and PLA was additionally tested by analyzing the co-hydrolysis of a fluorogenic probe (4-MUL) embedded in a polymer matrix made of pure PBAT, L-PLA, D-PLA, or L/D-PLA. Hydrolysis was measured using pre-cultures of the microbial strains cultured in MM containing ecovio^®^ at three different timepoints (t0–t2). Many microbial strains were able to co-hydrolyze PBAT and 4-MUL (Supplementary Figure S9). Most 4-MUL was hydrolyzed by fungal strains 737 (*Thelebolus globosus*), 800 (*Neodevriesia* sp.), 918 (*Penicillium stoloniferum*), 943 (*Lachnellula* sp.) and 1,031 (*Pseudogymnoascus verrucosus*), and by bacterial strains 780 (*Collimonas arenae*), 950 (*Streptomyces avidinii*) and 985 (*Amycolatopsis* sp.).

**Figure 5:**
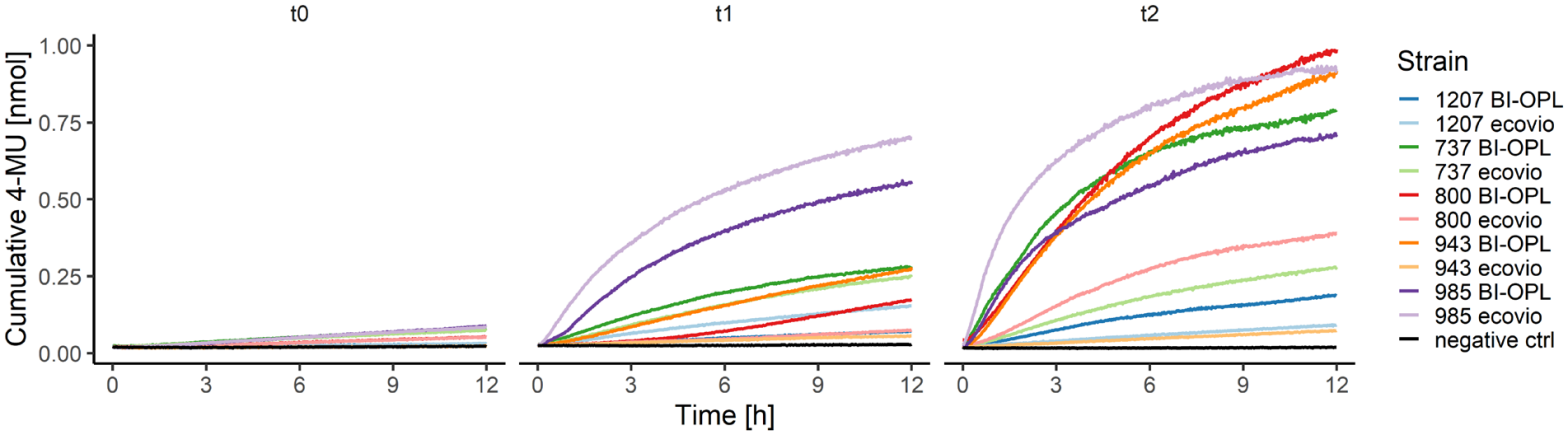
Cumulative 4-MU released by co-hydrolysis of 4-MUL and the polybutylene adipate-co-terephthalate (PBAT) matrix over time by selected microbial strains. BI-OPL-degrading strains were incubated with both ecovio^®^ and BI-OPL in mineral medium (MM). Subsamples were taken at different timepoints and added to the polymer-coated 96-well plates. The increase in fluorescence intensity was measured for 12 h and converted into cumulative 4-MU released with a calibration curve. t0, directly after incubation; t1, 7 days after incubation; t2, 25 days after incubation. Only the means of all three replicates per strain and medium are shown for ease of visual presentation. Microbial strains: 1207 (*Oidiodendron echinulatum*), 737 (*Thelebolus globosus*), 800 (*Neodevriesia* sp.), 943 (*Lachnellula* sp.), and 985 (*Amycolatopsis* sp.).

For the significant BI-OPL-degrading strains identified by weight-loss screening (Figure 2), pre-cultures were prepared for both plastics (ecovio^®^ and BI-OPL) for co-hydrolysis experiments of the pure polymers (PBAT and PLA) and 4-MUL. Levels of hydrolysis gradually increased from t0 to t2 for all strains and, except for strain 985 (*Amycolatopsis* sp.), higher levels of hydrolysis were detected when the plastic films were pre-cultured with BI-OPL compared with ecovio^®^ (Figure 5).

No degradation of L-PLA, D-PLA, or L/D-PLA was detected for any of the tested strains (Supplementary Data S3). However, proteinase K (Tokiwa et al., 2009) and the bacterial strain DSM 44262 (*Amycolatopsis alba*) (Butbunchu and Pathom-Aree, 2019) were also tested as positive controls for PLA degradation, and no increase in the fluorescence intensity was detected (Supplementary Figure S10). Therefore, we cannot definitively conclude that any of the tested microbial strains is able to degrade PLA under the tested conditions.

## 4. Discussion

### 4.1. Actinobacteria, Proteobacteria, and Ascomycota are dominant taxa in the plastisphere of terrestrial cryoenvironments

In our study we isolated microbial strains from the plastisphere of cold terrestrial environments belonging almost exclusively to the phyla Actinobacteria, Proteobacteria and Ascomycota. Especially the orders Burkholderiales, Pseudomonadales, and Streptomycetales and the genus *Pseudogymnoascus* were repeatedly isolated, which agrees with previous reports of the soil plastisphere microbiome analyzed by culture-independent genomic methods (Rüthi et al., 2020, 2023; Ju et al., 2021; Sun et al., 2022; Zhang et al., 2022) and microbial cultivation (Cosgrove et al., 2007). Further, these phyla (+ Firmicutes) are the principal taxa known to be involved in the degradation of PUR, PBAT and PLA (Cregut et al., 2013; Gambarini et al., 2021).

### 4.2. Cold-adapted bacteria and fungi are able to degrade dispersed PUR and polyester films

**Figure 6:**
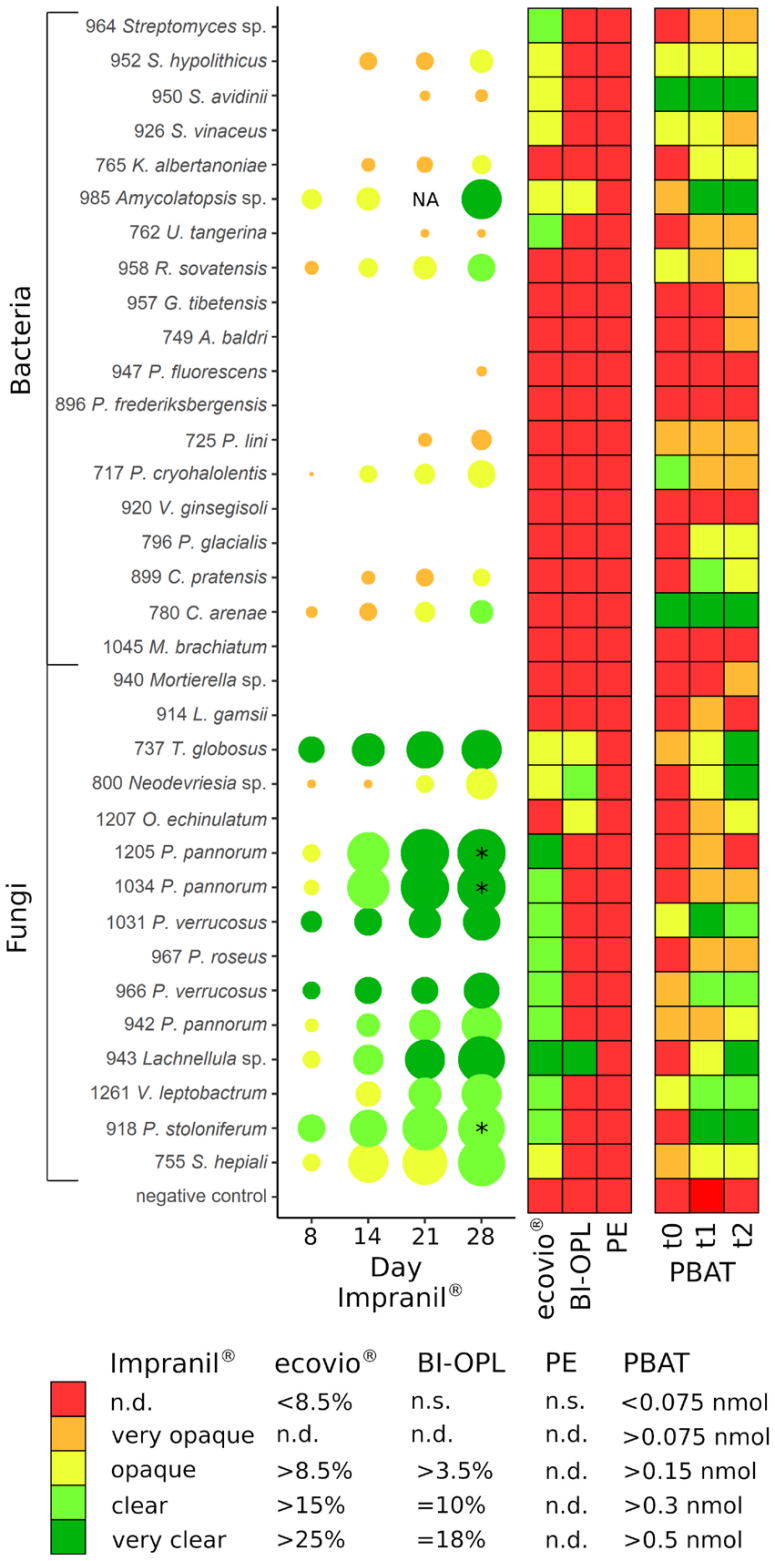
(Left) Halo formation over incubation time by microbial strains on plates with mineral medium plus Impranil^®^ (MM + Imp). The diameters of the circles represent the diameters of the halos relative to the colony size, and the color indicates the clearance of the agar on a given day. (Middle) Results of the plastic film degradation screening by weight loss (in %). (Right) Results of the 4-MUL assays using pure PBAT. PE, polyethylene; PBAT, polybutylene adipate-co-terephthalate; NA, not analyzed (for isolate 985 no photo was taken after 21 days); * halo diameter influenced by the microbial strains spreading on the agar plate by spores; n.s., not significant; n.d., not defined. For strains 737 (*Thelebolus globosus*), 800 (*Neodevriesia* sp.), 943 (*Lachnellula* sp.), 985 (*Amycolatopsis* sp.), and 1,207 (*Oidiodendron echinulatum*) the results of pre-cultures with BI-OPL films are shown in PBAT t0 – t1, whereas for all other strains the results of pre-cultures with ecovio^®^ films are shown. For strains 952, 950, and 926: *S.*, *Streptomyces*, *K*., *Kribbella*, *U*., *Umezawaea*, *R*., *Rhodococcus*, *G*., *Glaciihabitans*, *A*., *Agrococcus*; for strains 947 and 896: *P*., *Pseudomonas*; for strain 717: *P*., *Psychrobacter*, *V*., *Variovorax*; for strain 796: *P*., *Polaromonas*, *C*., *Collimonas*, *M*., *Methylobacterium*, *L*., *Linnemannia*, *T*., *Thelebolus*, *O*., *Oidiodendron*; for strains 1,205, 1,034, 1,031, 967, 966, and 942: *P*., *Pseudogymnoascus*, *V*., *Verticillium*; for strain 918: *P*., *Penicillium*; for strain 755: *S*., *Samsoniella*. Detailed information on each microbial strain is given in Table 1.

The abilities of the microbial strains from alpine and Arctic habitats to degrade dispersed PUR; and PBAT- and PLA-based polyester films at low temperatures (15°C) were summarized in Figure 6. Even though cold-adapted microbial strains often thrive at temperatures below 15°C, it has been reported that maximum enzymatic activity of psychrophilic and psychrotolerant microorganisms is above their upper growth limit (Huston et al., 2000). Temperature optima for growth, enzyme production and enzyme activity are relevant factors for plastic degradation and need testing in further studies. Our agar plate clearing experiment showed that more than half of the tested isolates had the potential to degrade Impranil^®^, a PUR dispersion. An earlier study indicated that approximately 30% of fungi isolated from plastic litter floating in a lake were Impranil^®^ degraders (Brunner et al., 2018). Urbanek et al. (2017) showed degradation of at least one of the tested biodegradable plastics by around 39% of microbial strains isolated from Arctic environments using agar clearing assays with polycaprolactone, polybutylene succinate and polybutylene succinate-co-adipate at 28°C. Especially members of the Ascomycota proved to be efficient PUR degraders at low temperatures (15°C) in our study. In accordance with our findings, Barratt et al. (2003) observed that PUR degradation in soil was driven primarily by fungi. PUR degradation was previously reported for the genera *Penicillium* (Brunner et al., 2018), *Pseudogymnoascus* (synonym *Geomyces*; Barratt et al., 2003; Cosgrove et al., 2007), *Pseudomonas* (Cregut et al., 2013), *Rhodococcus* (Akutsu-Shigeno et al., 2006), and *Verticillium* (Navarro et al., 2021). However, to our knowledge this is the first report of Impranil^®^ degradation by the bacterial genera *Amycolatopsis*, *Collimonas*, *Kribbella*, *Psychrobacter,* and *Streptomyces* and by the fungal genera *Lachnellula*, *Neodevriesia,* and *Thelebolus*.

We further identified 12 microbial strains with the ability to significantly break down ecovio^®^ and five able to degrade BI-OPL at 15°C. Both of these products are commercial materials made of PBAT and PLA, as well as other, unknown components and additives. Hence, we cannot conclude depolymerization of one of those polymers solely from total weight loss. However, testing degradation of commercial plastic products is relevant because in reality commercial plastics, rather than pure polymers, are released to the environment and recycled. Therefore, suitable microorganisms and enzymes would need to have the ability to degrade solid, commercial products consisting of synthetic polymer components as well as other components and additives in order to be useful for remediation and recycling applications. Interestingly, degradation by cold-adapted microorganisms has primarily been found for polycaprolactone so far (Sekiguchi et al., 2011; Urbanek et al., 2017, 2018, 2021). For instance, Urbanek et al. (2017) reported a 34.5% weight loss of polycaprolactone films when incubated with an Arctic *Clonostachys rosea* strain for 1 month at 21°C. In addition, the same authors reported degradation of polycaprolactone (6%) and poly(butylene succinate-co-butylene adipate) (26%) by a *Pseudogymnoascus* strain from Antarctic soil within 1 month at 14°C (Urbanek et al., 2021), which is comparable to some of our strains with ecovio^®^ and BI-OPL films at 15°C. Degradation of PLA and PBAT has, so far, only been found for mesophilic and thermophilic microbial strains. Nakajima-Kambe et al. (2009) reported degradation of plastic films made of ecoflex^®^ (i.e., PBAT) by *Leptothrix*, a taxon not isolated in our study, at 30°C. In another study, PBAT films lost 9% of their weight after incubation at 25°C for 20 days with *Rhodococcus fascians* (Soulenthone et al., 2020). In our study a strain of the same genus (958 *Rhodococcus sovatensis*) did not significantly reduce the weight of the plastic films, but it was able to hydrolyze PBAT in the 4-MUL assay and to degrade Impranil^®^. In previous studies, evidence for PLA degradation has mostly been provided for the phylum Actinobacteria, including the genera *Amycolatopsis*, *Streptomyces*, and *Saccharothrix*, and at temperatures of 30°C or higher (Jarerat et al., 2002; Jarerat and Tokiwa, 2003; Tokiwa and Jarerat, 2005; Sriyapai et al., 2018). In our study the strains 964 (*Streptomyces* sp.) and 985 (*Amycolatopsis* sp.) significantly reduced the weight of ecovio^®^ and BI-OPL films, respectively. Whereas 964 (*Streptomyces* sp.) only degraded ecovio^®^ components other than PBAT and PLA, 985 (*Amycolatopsis* sp.) was able to degrade the PBAT and PLA components of the BI-OPL film. Our results indicate that members of these genera also have the potential to degrade plastics at low temperatures (15°C). Strain 762 (*Umezawaea tangerina*) significantly reduced the weight of ecovio^®^. When cultured in R2A, it significantly degraded the PLA component of the plastic films. *Umezawaea* has not previously been shown to degrade plastics, but it is closely related to the PLA-degrading genus *Saccharothrix* (Labeda and Kroppenstedt, 2007). While strain 762 was isolated from plastic collected in Svalbard, in an earlier investigation we also found enrichment of the genus in the plastisphere of biodegradable plastics in alpine soil (Rüthi et al., 2020).

The fungal genus *Pseudogymnoascus* is abundant in soils of cold environments and was shown to be enriched in the plastisphere of biodegradable plastics in alpine and Arctic soils (Rüthi et al., 2020). All of the *Pseudogymnoascus* isolates tested here significantly reduced the weight of ecovio^®^. Interestingly, strains of the *Pseudogymnoascus* species *P. pannorum* and *P. verrucosus* were able to degrade Impranil^®^, whereas the only strain of *P. roseus* (strain 967) was not. Furthermore, *P. roseus* did not degrade the PBAT and PLA components of ecovio^®^. Other *Pseudogymnoascus* strains degraded the PBAT (1,034 and 1,205 *P. pannorum*, 966 and 1,031 *P. verrucosus*) and the PLA (1,031 *P. verrucosus*) components of the ecovio^®^ films. Urbanek et al. (2021) recently reported degradation of polycaprolactone and poly(butylene succinate-co-butylene adipate) at 14°C by an Antarctic *Pseudogymnoascus* strain. Notably, the plastic degradation rate by this strain was higher at 14°C than at 20°C. They identified two enzymes produced by the *Pseudogymnoascus* strain with the ability to degrade several biodegradable polyesters (Urbanek et al., 2022). Taken together, these results indicate that the ability to degrade polyester-type polymers at low temperatures is widespread in the genus *Pseudogymnoascus*.

The fungal strains 737 (*Thelebolus globosus*), 918 (*Penicillium stoloniferum*), 1,207 (*Oidiodendron echinulatum*), 800 (*Neodevriesia* sp.), and 943 (*Lachnellula* sp.) were able to degrade at least one of the plastic films. The last two strains were able to reduce the weight of both ecovio^®^ and BI-OPL, and both strains significantly degraded the PBAT and PLA components of the plastic films. Strain 737 (*Thelebolus globosus*) significantly degraded the PBAT and PLA in BI-OPL films when cultured in R2A and the PBAT when cultured in MM. *Thelebolus* is a psychrophilic fungal genus known to thrive in Antarctic lakes (de Hoog et al., 2005). It has been shown to produce extracellular enzymes (α-amylase) with a maximum activity at 20°C and rapidly decreasing activity at higher temperatures, which emphasizes its adaptation to cold environments (Singh et al., 2014). *Lachnellula* comprises both saprotrophic and plant-pathogenic species, causing, e.g., larch canker (Giroux and Bilodeau, 2020). Plant-pathogenic species have often been reported to degrade polyesters because of the ability to produce cutinases that target the polymers, due their resemblance to the plant polymeric substance cutin (Brodhagen et al., 2015). To our knowledge, this is the first report of *Thelebolus* and *Lachnellula* strains in the context of plastic degradation. The genus *Neodevriesia* is found in a broad range of habitats (Wang et al., 2017). Recently, a *Neodevriesia* strain with the ability to form halos on agar containing polycaprolactone was isolated from a sea coast (Kim et al., 2022). The genus *Oidiodendron* has been found to be enriched in the plastisphere of biodegradable plastics in alpine soil (Rüthi et al., 2020). This genus mostly inhabits soil and decaying plant material, and some species have been shown to produce enzymes, including lipases, gelatinases and polyphenol oxidases (Rice and Currah, 2005). *Oidiodendron* has also been observed to be involved in the deterioration of natural and synthetic rubber (Lugauskas et al., 2021). *Penicillium* strains are well-known for their broad metabolic capabilities, including degradation of various plastic types (Srikanth et al., 2022).

The isolated cold-adapted microorganisms are not only valuable for plastic degradation in the environment, but could also prove useful for recycling or upcycling by producing plastic-degrading enzymes active at lower temperatures. However, it needs to be stated that the tested strains required a long time to degrade the plastic films (analyzed after 60 and 126 days). In addition, whereas other studies reported almost complete degradation of plastic films (Tokiwa and Jarerat, 2005), the largest weight loss achieved in our study was 46% by strain 1,205 (*Pseudogymnoascus pannorum*) for ecovio^®^ and 39% by strain 737 (*Thelebolus globosus*) for BI-OPL after 60 days. The incomplete degradation might be due to too short incubation periods, due to the inability to degrade specific components of the films, or due to the material properties of the films (e.g., crystallinity). Semi-crystalline polymers, such as PBAT, contain crystalline domains that are more recalcitrant than the amorphous domains (Tokiwa et al., 2009). Interestingly, we observed a strong linear correlation between the PBAT mass loss and terephthalate content in both biodegradable plastic films, which, since enzymes preferentially attack butylene adipate-rich components of PBAT, supports that the films were enzymatically degraded. Increased ratios of aromatic to alipathic groups in PBAT were previously shown to slow down hydrolysis by enzymes (Zumstein et al., 2017b) and might also be a reason for the incomplete degradation of plastic films in our experiments. Other explanations could be accumulation of waste products, depletion of essential nutrients, or acidification of the culturing medium, all of which could inhibit microbial growth and plastic degradation at some point.

Unsurprisingly, we did not find microbial strains able to degrade PE in the tested timeframe. In most other studies, microbial strains partially degraded artificially weathered (e.g., by UV radiation) PE, and only a few were demonstrated to affect material properties of untreated PE (Yang et al., 2014; Sowmya et al., 2015). Such pretreatments can oxidize PE, thereby making it more biodegradable (Mohanan et al., 2020). Even though some studies have shown indications of PE degradation by microorganisms, this polymer is broadly considered non-biodegradable under environmental conditions.

### 4.3. Co-hydrolysis of polymer-embedded 4-MUL shows the ability of microbial strains to hydrolyze PBAT

We identified a large number of strains with the ability to hydrolyze PBAT using the 4-MUL assay. Generally, the results of the fluorescence-based method correlated well with the Impranil^®^ plate clearing assays, weight-loss screening and NMR for many strains (Figure 6). For instance, we detected a strong release of 4-MU for the BI-OPL- and Impranil^®^-degrading strains 737 (*Thelebolus globosus*), 800 (*Neodevriesia* sp.), 943 (*Lachnellula* sp.), and 985 (*Amycolatopsis* sp.). Strikingly, 4-MUL hydrolysis gradually increased over the three measured timepoints for these strains, suggesting that they produced more PBAT-degrading enzymes over time while living with BI-OPL as the sole C source. For most ecovio^®^-degrading strains we also detected 4-MUL hydrolysis, suggesting that the PBAT component of the plastic films was degraded by the microorganisms, although there were some exceptions. For example, for strains 964 (*Streptomyces* sp.) and 967 (*Pseudogymnoascus roseus*) we detected only very low levels of 4-MUL hydrolysis over the entire 28-day period. These findings support the results of the NMR analysis suggesting that both strains only degraded components of the ecovio^®^ films other than PBAT and PLA. For strain 1,205 (*Pseudogymnoascus pannorum*) we found low levels of 4-MUL hydrolysis, but NMR analysis indicated significant reduction in the mass of the PBAT fraction of the plastic films for strain 1,205 (*Pseudogymnoascus pannorum*). Possible explanations for this discrepancy are that the material properties of the PBAT in the ecovio^®^ and the pure PBAT used in the 4-MUL experiment were different. For example, molecular weight, monomer composition, crystallinity and surface structure all influence the hydrolyzability of plastics (Tokiwa et al., 2009; Zumstein et al., 2017b; Mohanan et al., 2020), which could have impacted our findings. It is further conceivable that potential unfavorable culture conditions in the 4-MUL assays could have inhibited the expression of PBAT-degrading enzymes. On the contrary, we also detected 4-MUL hydrolysis for strains that did not significantly reduce the weight of the plastic films. For most strains only small amounts of 4-MU were released, and the discrepancy between the two assays was likely due to higher sensitivity of the fluorescence-based approach. Such low levels of 4-MUL hydrolysis may be due to enzymes with very low activity or hydrolyzing only amorphous domains of PBAT, not resulting in a significant weight loss. However, strains 950 (*Streptomyces avidinii*) and 780 (*Collimonas arenae*) showed high levels of PBAT hydrolysis over the entire 28 days, providing evidence that they constitutively expressed an efficient PBAT-degrading esterase over a long period. It might be that the PBAT esterases produced by these strains were active at higher temperatures (30°C in the 4-MUL assays) but not at low temperatures (15°C in the weight-loss assay). Some *Streptomyces* strains are able to degrade PLA and other polyesters (Sriyapai et al., 2018), but PBAT degradation has not been reported previously. *Collimonas* has been found to be enriched in the plastisphere of biodegradable plastics in alpine soil (Rüthi et al., 2020). This genus is known for mycophagy and weathering capabilities (Frey et al., 2010; Leveau et al., 2010), and in the genomes of *Collimonas* strains many genes encoding extracellular enzymes, such as chitinases and proteases, have been found (Song et al., 2015). However, the genus has not been reported to degrade plastics. Even though these strains did not significantly reduce the weight of the plastic films in the initial screening phase of our study, they are promising candidates for PBAT degradation and further research is needed to better understand the factors affecting plastic degradation by these strains. We acknowledge that the 4-MUL assay only shows a short snapshot, whereas the weight-loss method detects degradation over a long period and potentially different growth phases of microorganisms. Weight-loss measurements, however, cannot provide direct information on polymer hydrolysis. Therefore, testing multiple timepoints and applying several methods to verify plastic degradation are essential for obtaining detailed insights.

### 4.4. The composition of the culturing medium affects microbial strains in different manners

We found that the composition of the culturing medium had a highly significant impact on the degradation of ecovio^®^ and BI-OPL films. However, the microbial strains did not follow a general trend. Strains 943 (*Lachnellula* sp.) and 1,205 (*Pseudogymnoascus pannorum*) were most successful in degrading the plastic films when cultured in MM medium containing no C source except the plastics. For strain 1,205 (*Pseudogymnoascus pannorum*) we further found that the PBAT in the ecovio^®^ films was only significantly degraded when the strain was cultured in MM. This suggests that these strains selectively secreted enzymes to degrade at least one component of the plastics when no other, more readily available C source was present. For strains 737 (*Thelebolus globosus*) and 762 (*Umezawaea tangerina*) the largest total weight loss and significant mass reduction of the PLA component occurred when R2A was used as the culturing medium. The same trend was observed for the other actinobacterial isolates, 985 (*Amycolatopsis* sp.) and 964 (*Streptomyces* sp.). There may be an initial need for a C and energy source in order to produce plastic-degrading enzymes, or a component of the R2A may induce expression of polymer-degrading enzymes in these strains. Another explanation might be that these strains accumulated faster in the R2A medium, resulting in more cells producing plastic-degrading enzymes in the tested timeframe. The effect of the culture medium composition on the degradation of plastics has been tested in surprisingly few studies (Watanabe et al., 2014; Sriyapai et al., 2018). Addition of gelatin and similar substances has been observed to stimulate extracellular protease expression and consequently PLA degradation by bacteria, including *Amycolatopsis* spp., and fungi (Jarerat and Tokiwa, 2001; Tokiwa and Calabia, 2006). In contrast to these reports, the addition of gelatin to the culture medium decreased the degradation of plastics by most strains in our study. Only for strain 800 (*Neodevriesia* sp.) was the degradation of plastic films induced by the addition of gelatin to both MM and R2A. However, analysis by NMR showed no increased degradation of PLA by addition of gelatin for this strain. These results demonstrate that the composition of the culture medium has a large influence on the extent of degradation and that there is no culture medium working best for all strains. Consequently, it is likely that screening tests for plastic-degrading microorganisms often only detect a subset of the potential plastic-degrading strains because only a few conditions are tested, whereas some strains may require very specific conditions to express plastic-degrading enzymes. A promising method to optimize plastic degradation might involve the identification of the genes encoding the responsible enzymes and the heterologous expression of these genes in a suitable host (Perfumo et al., 2020).

## 5. Conclusion

In this study we analyzed the potential of microbial strains isolated from the plastisphere of cold terrestrial environments to degrade different plastics. Several taxa (e.g., genera *Collimonas*, *Kribbella*, *Lachnellula*, and *Thelebolus*) were shown, for the first time, to degrade plastics. Most notably, the tested strains degraded dispersed PUR and the polyester films ecovio^®^ and BI-OPL at lower temperatures (15°C) than previously reported microbial strains. The fungal strains 800 (*Neodevriesia* sp.) and 943 (*Lachnellula* sp.) are promising candidates for further studies, as they degraded all the tested biodegradable products, were shown to reduce the masses of the PBAT and PLA components in the plastic films, and efficiently hydrolyzed the pure PBAT polymer. In addition, we demonstrated that culturing conditions have a strong influence on plastic degradation. This finding might help to optimize the degradation rates achieved by the microbial strains and may also have consequences for plastic degradation in natural environments where carbon and nutrient contents are limited, in particular in oligotrophic Arctic and high-mountain soils. This study expands our knowledge about microbial plastic degradation and provides a basis for future discovery of cold-active plastic-degrading enzymes. The identified microbial strains could serve as a valuable resource for the development of efficient and sustainable plastic-waste recycling at lower temperatures.

## Data availability statement

The datasets presented in this study can be found in online repositories. The names of the repository/repositories and accession number(s) can be found in the article/Supplementary material.

## Author contributions

BF and JR designed the study. BF collected the samples. BS, IB, and JR isolated and characterized the microbial strains. IB and JR performed the clear zone assays. BF, BS, and JR performed the weight-loss screenings and data analysis. JR, MC, and MS performed the NMR and 4-MUL assays and data analysis. JR wrote the manuscript with input from BF, IB, and MS. All authors contributed to the article and approved the submitted version.

## Funding

This work was supported by two WSL internal grants (5231.00900.002.01, Metagenomics and 5233.00388.001.01, Bioactive permafrost). Open access funding by Swiss Federal Institute for Forest, Snow and Landscape Research (WSL).

## Conflict of interest

MS and MC declare collaboration with BASF SE on other projects related to polymer biodegradation and MS for receiving funding for these projects from BASF SE.

The remaining authors declare that the research was conducted in the absence of any commercial or financial relationships that could be construed as a potential conflict of interest.

## Publisher’s note

All claims expressed in this article are solely those of the authors and do not necessarily represent those of their affiliated organizations, or those of the publisher, the editors and the reviewers. Any product that may be evaluated in this article, or claim that may be made by its manufacturer, is not guaranteed or endorsed by the publisher.
