# Supplementary material for: Discovery of plastic-degrading microbial strains isolated from the alpine and Arctic terrestrial plastisphere
Source: Front Microbiol. 2023 May 10;14:1178474. doi: 10.3389/fmicb.2023.1178474 (PMC10206078; doi:10.3389/fmicb.2023.1178474)
Supplement: Supplementary file 1 [file data_sheet_1.docx]

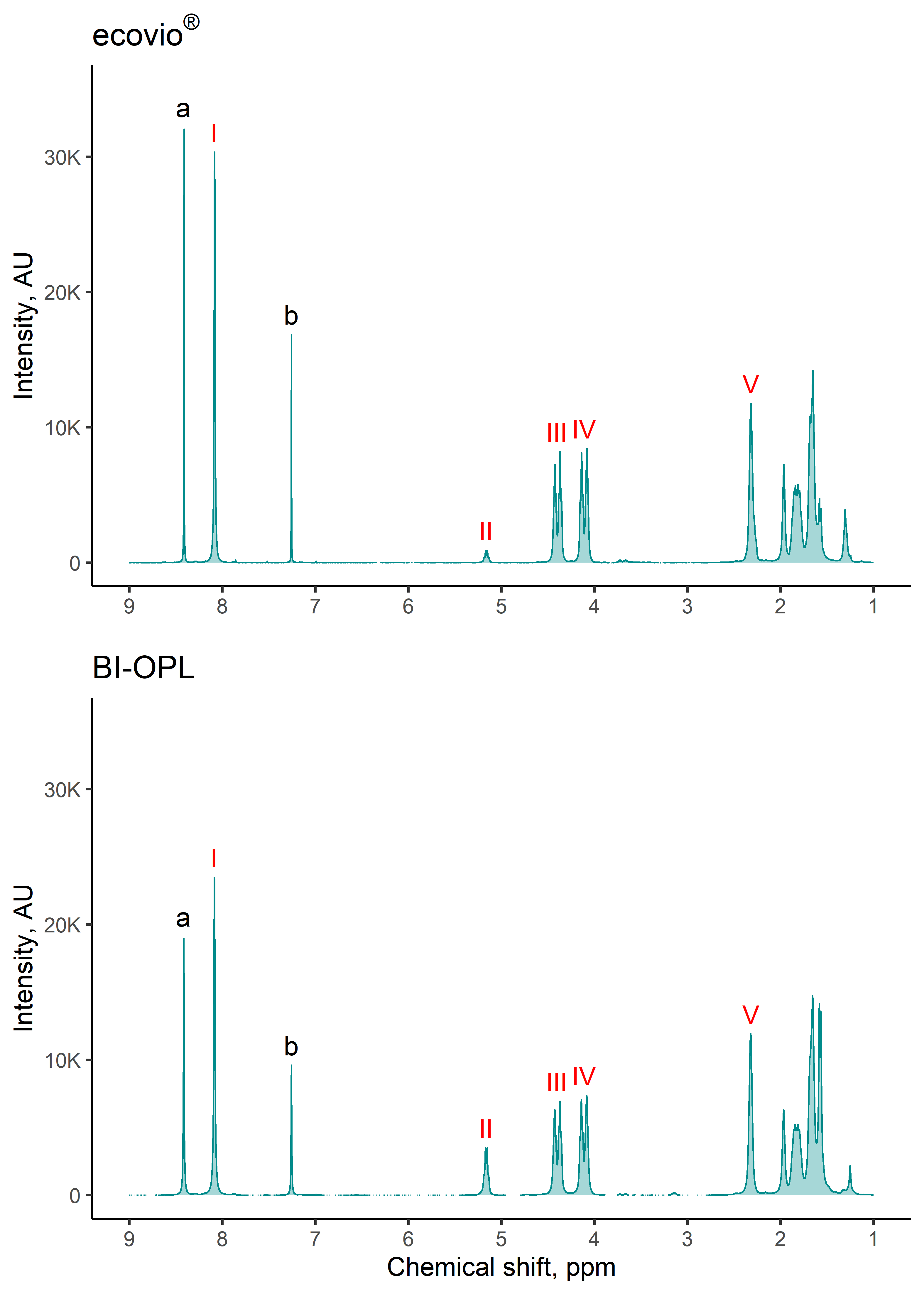


**Figure S1:** Annotated ^1^H-NMR spectra of the of poly(butylene adipate-co-terephthalate) (PBAT) and polylactic acid (PLA) components contained in the commercial mulch films ecovio^®^ (top) and BI-OPL (bottom). The spectra were collected in deuterated chloroform, using 1,4-dinitrobenzene (DNB) as internal standard for quantification of the polymer components. Peaks I:V correspond to the polymer signals, peak (a) to the internal standard and peak (b) to the traces on non-deuterated solvent. All chemical structures and the identities of the protons are reported in Table S6.


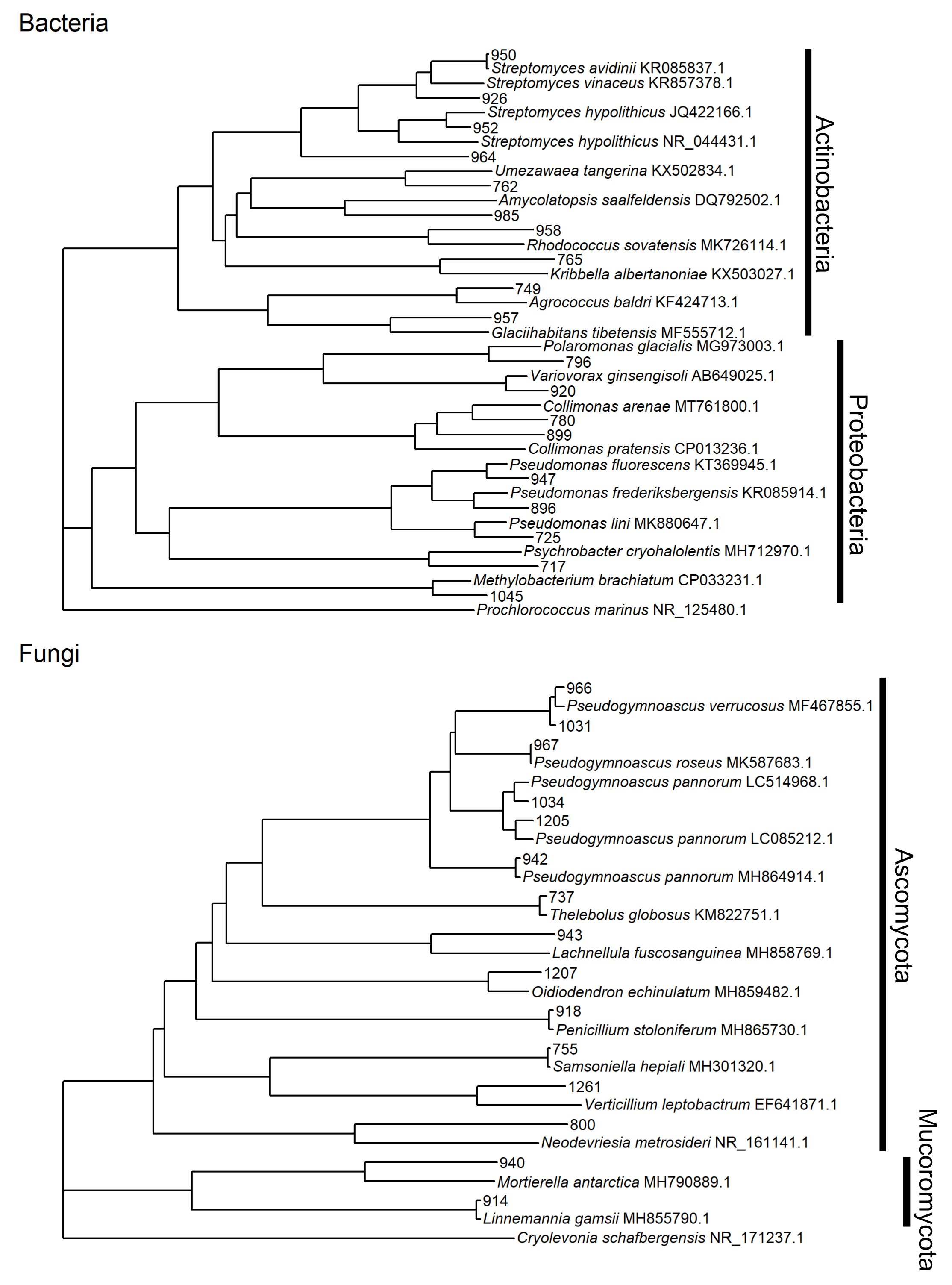


**Figure S2:** Phylogenetic tree of the microbial strains isolated from the plastisphere and their closest related species determined by BLASTn. Reference sequences were retrieved from the NCBI nucleotide database. Species names and NCBI accession numbers are given. The trees were rooted using the NCBI sequences NR_125480.1 *Prochlorococcus marinus* (Cyanobacteria) for bacteria and NR_171237.1 *Cryolevonia schafbergensis* (Basidiomycota) for fungi as outgroups. Information about the microbial strains is given in Table 1.


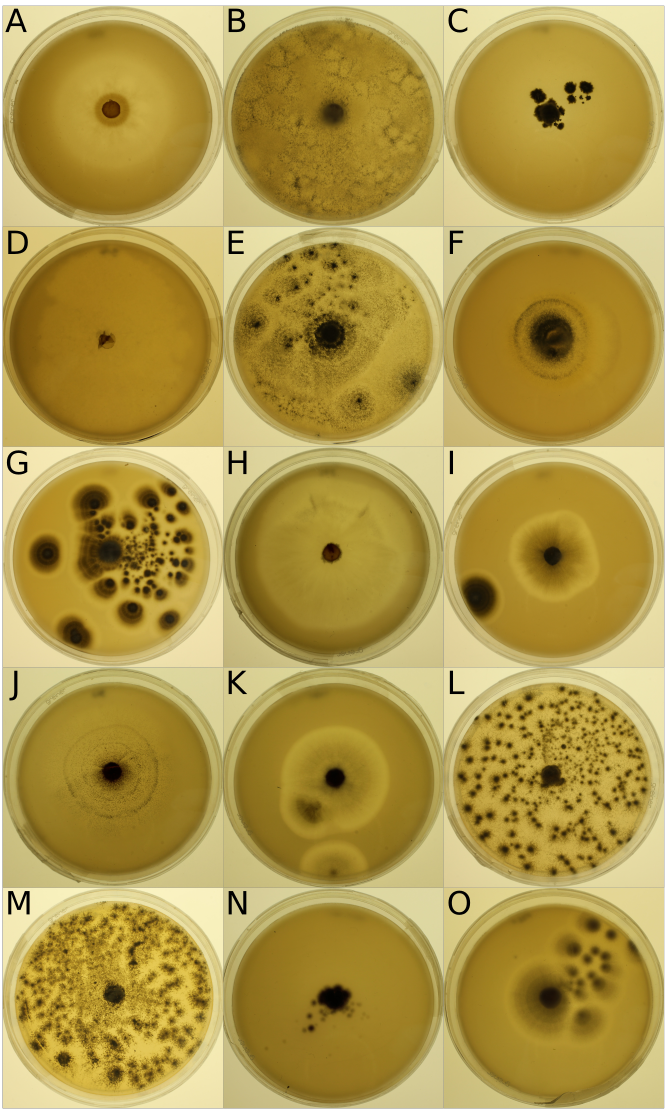


**Figure S3:** Impranil^®^ degradation by fungal strains after 28 days at 15°C. **(A)** 737 (*Thelebolus globosus*), **(B)** 755 (*Samsoniella hepiali*), **(C)** 800 (*Neodevriesia* sp.), **(D)** 914 (*Linnemannia gamsii*), **(E)** 918 (*Penicillium stoloniferum*), **(F)** 940 (*Mortierella* sp.), **(G)** 942 (*Pseudogymnoascus pannorum*), **(H)** 943 (*Lachnellula* sp.), **(I)** 966 (*Pseudogymnoascus verrucosus*), **(J)** 967 (*Pseudogymnoascus roseus*), **(K)** 1031 (*Pseudogymnoascus verrucosus*), **(L)** 1034 (*Pseudogymnoascus pannorum*), **(M)** 1205 (*Pseudogymnoascus pannorum*), **(N)** 1207 (*Oidiodendron echinulatum*), and **(O)** 1261 (*Verticillium leptobactrum*).


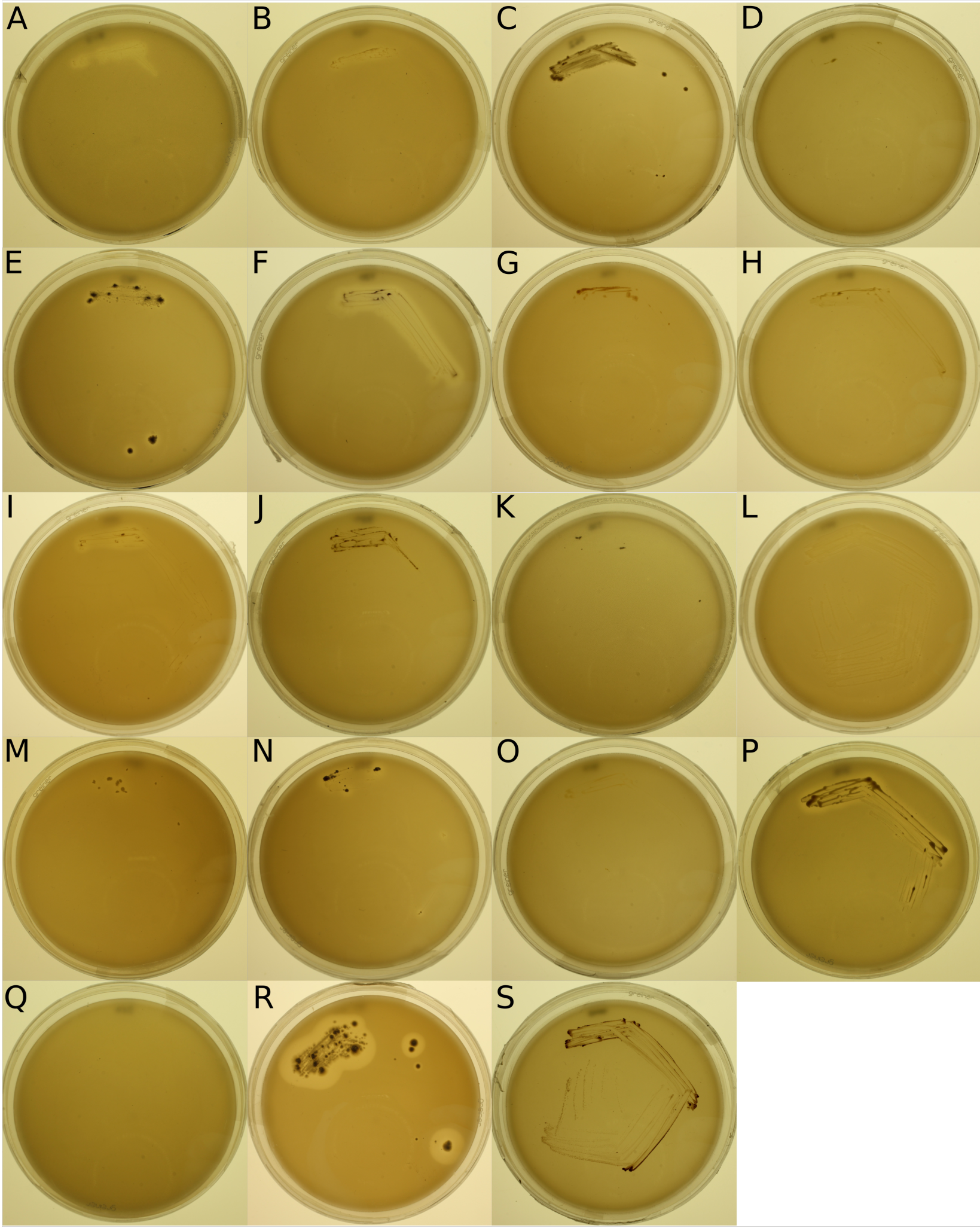


**Figure S4:** Impranil^®^ degradation by bacterial strains after 28 days at 15°C. **(A)** 717 (*Psychrobacter cryohalolentis*), **(B)** 725 (*Pseudomonas lini*), **(C)** 762 (*Umezawaea tangerina*), **(D)** 749 (*Agrococcus baldri*), **(E)** 765 (*Kribbella albertanoniae*), **(F)** 780 (*Collimonas arenae*), **(G)** 796 (*Polaromonas glacialis*), **(H)** (*Pseudomonas frederiksbergensis*), **(I)** 899 (*Collimonas pratensis*), **(J)** 920 (*Variovorax ginsegisoli*), **(K)** 926 (*Streptomyces vinaceus*), **(L)** 947 (*Pseudomonas fluorescens*), **(M)** 950 (*Streptomyces avidinii*), **(N)** 952 (*Streptomyces hypolithicus*), **(O)** 957 (*Glaciihabitans tibetensis*), **(P)** 958 (*Rhodococcus sovatensis*), **(Q)** 964 (*Streptomyces* sp.), **(R)** 985 (*Amycolatopsis* sp.), and **(S)** 1045 (*Methylobacterium brachiatum*).


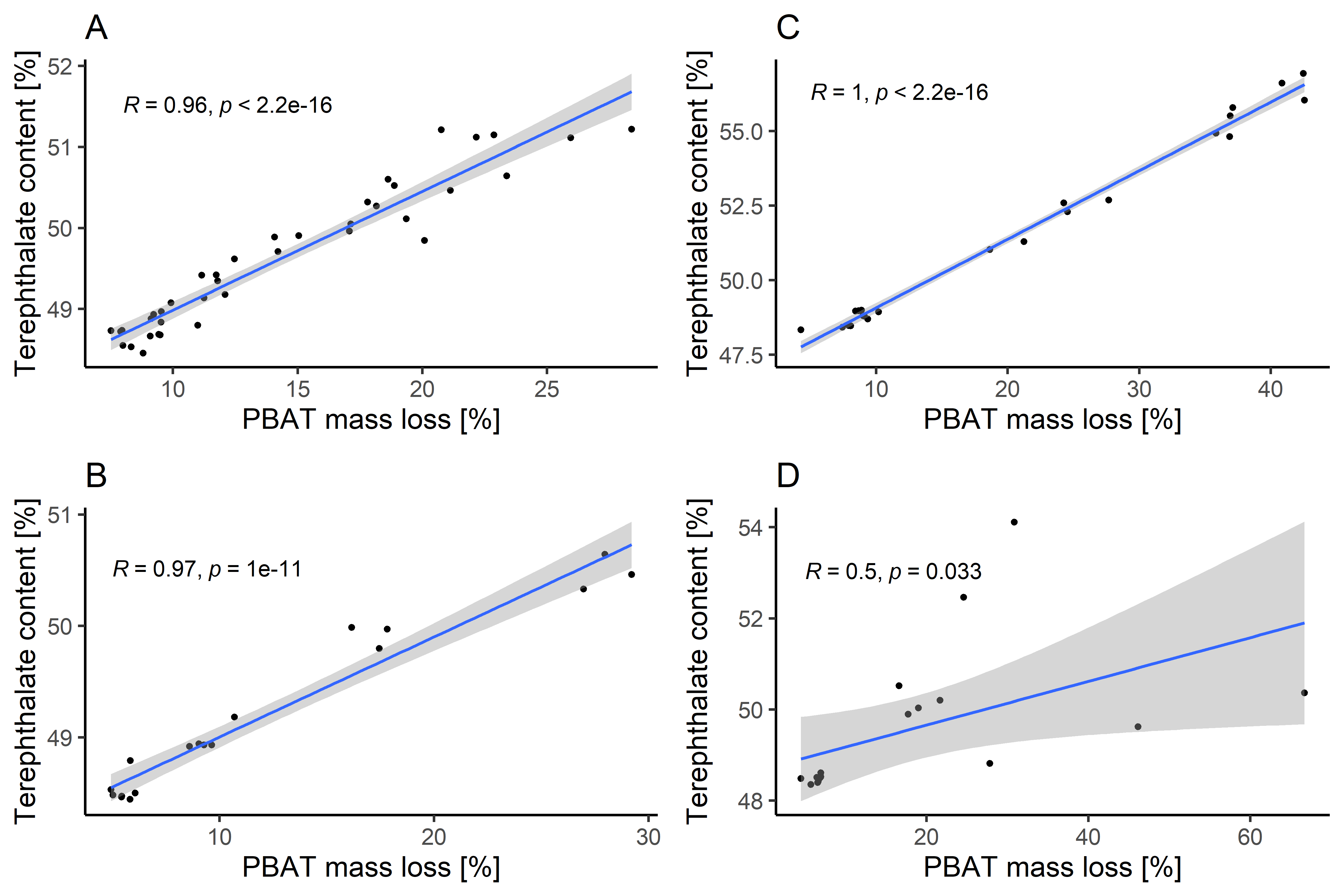


**Figure S5:** Linear correlation (Pearson) between the terephthalate content and mass loss of PBAT in the plastic films determined by NMR. Dots indicate samples, the blue lines represent the linear regression models and the grey areas show the confidence interval (95%). *R*- and *P*-values of the linear regression are given in each panel. **(A)** ecovio^®^ and **(B)** BI-OPL samples in section 3.3 (initial screening); **(C)** ecovio^®^ and **(D)** BI-OPL samples in section 3.4 (screening culturing medium).


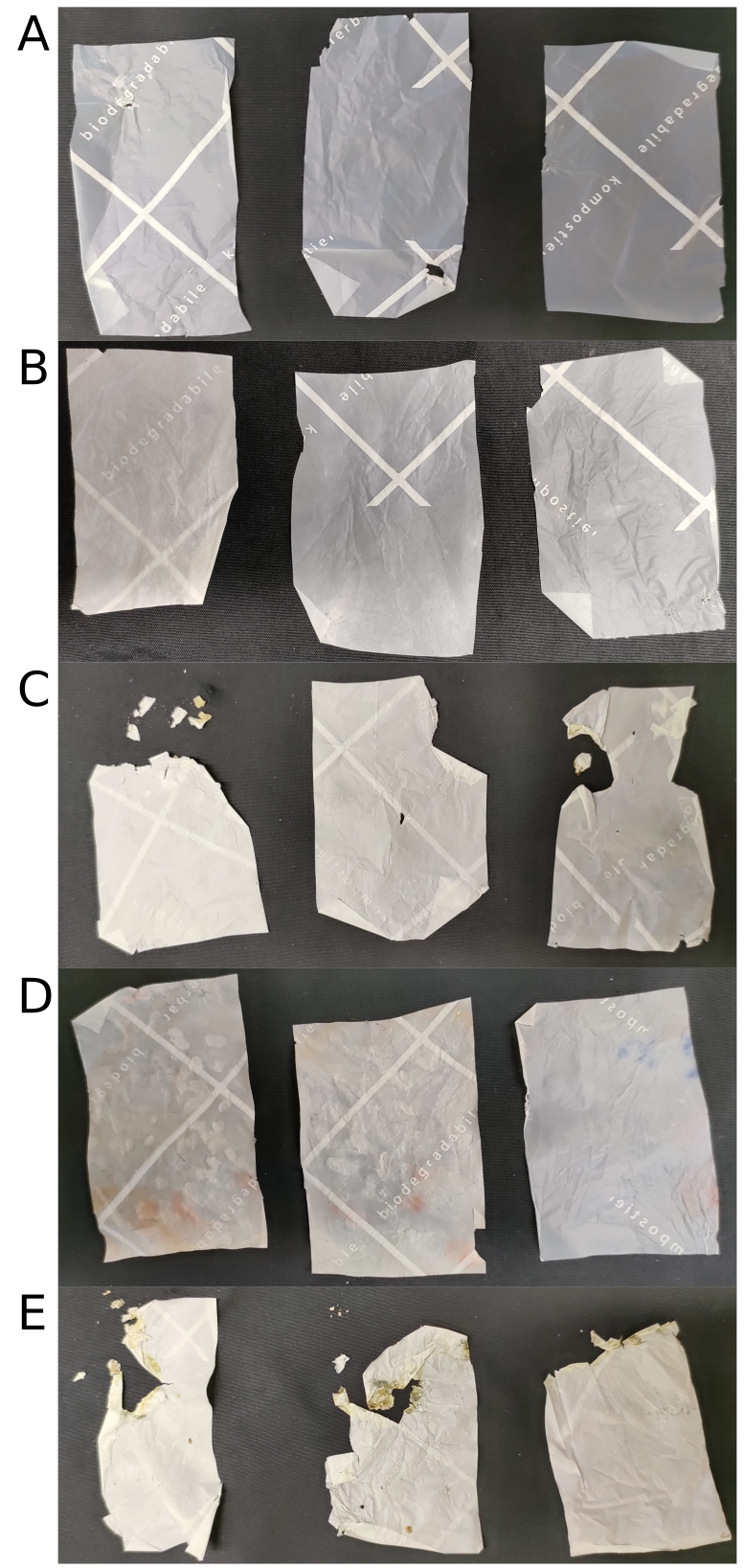


**Figure S6:** Examples of ecovio^®^ films after incubation with microbial strains in the initial weight-loss screening. All three replicates are shown for: **(A)** negative controls, **(B)** 762 (*Umezawaea tangerina*), **(C)** 943 (*Lachnellula* sp.), **(D)** 964 (*Streptomyces* sp.), and **(E)** 1205 (*Pseudogymnoascus pannorum*).


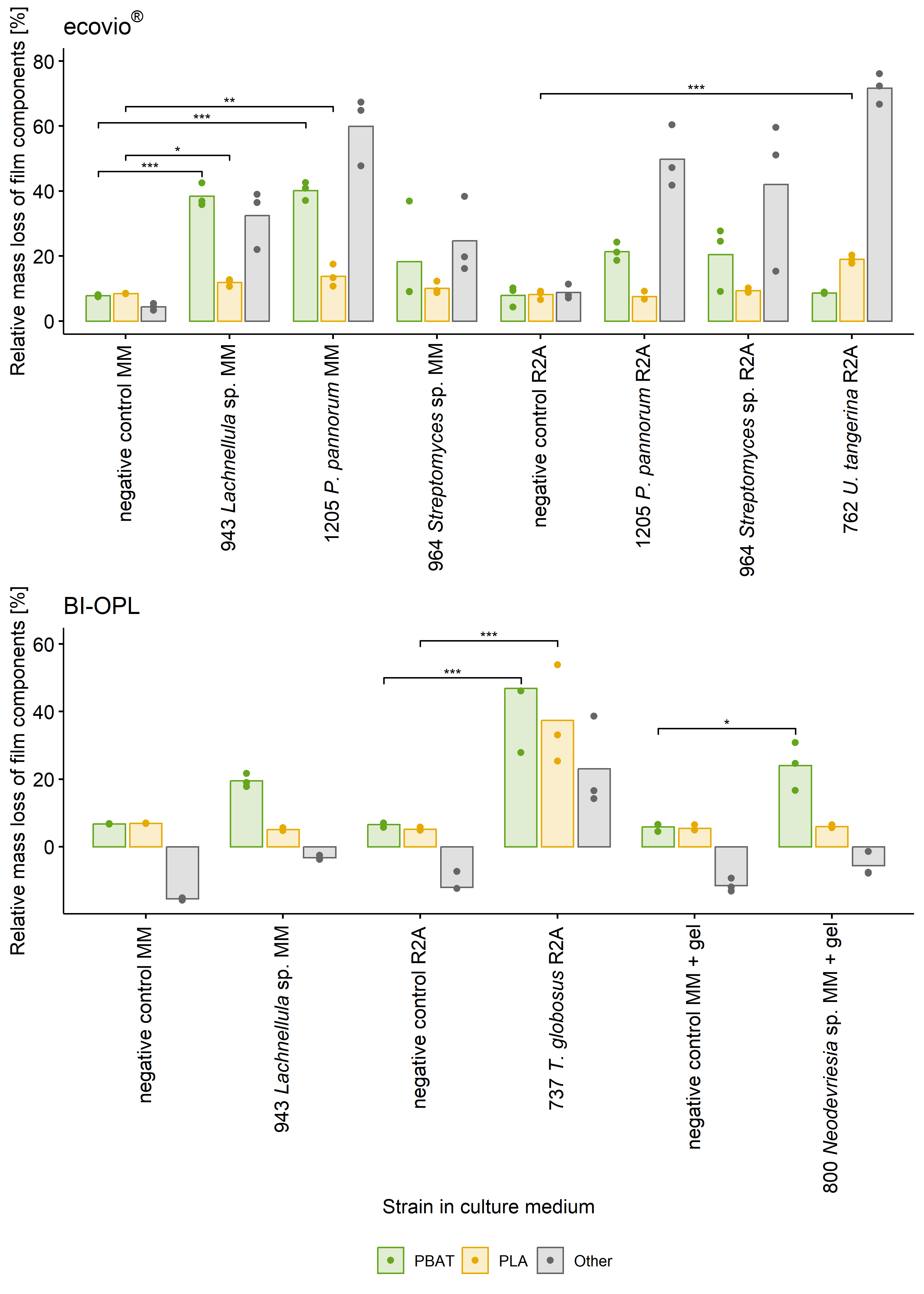


**Figure S7:** Mass loss of individual components (PBAT, PLA and other) in ecovio^®^ (top) and BI-OPL (bottom) films determined by NMR. Only strains with total weight losses significantly different from the negative controls were analyzed. Dots indicate the mass loss of the samples and bars indicate the means of three replicates. Colors indicate the individual film components. Asterisks indicate the level of significance between the negative controls and the strains, with *** *P* < 0.001, ** *P* < 0.01, and * *P* < 0.05. No statistical analysis was performed for components other than PBAT and PLA. *P*. = *Pseudogymnoascus*, *U*. = *Umezawaea*, *T*. = *Thelebolus*. Detailed information on each microbial strain is given in Table 1.


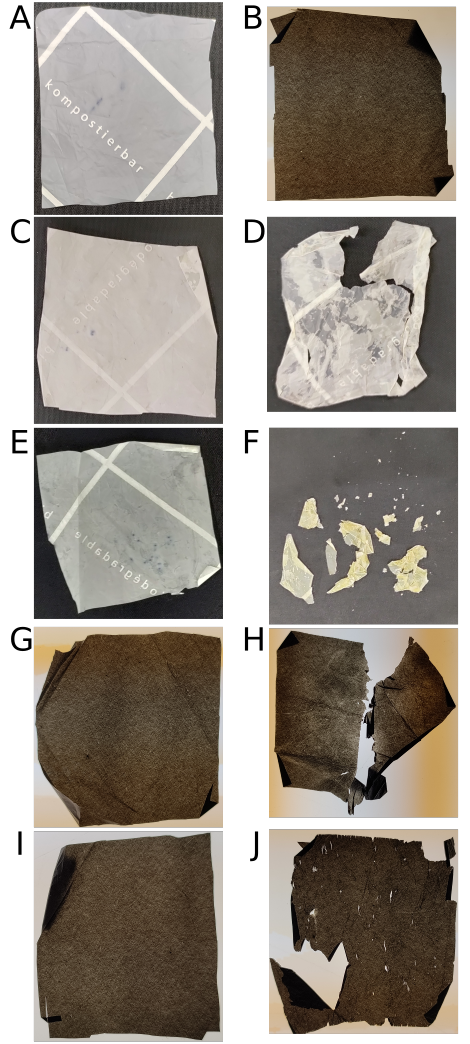


**Figure S8:** Examples of ecovio^®^ and BI-OPL films after incubation with microbial strains in different culturing media. Single replicates are shown for: **(A)** negative control ecovio^®^ in mineral medium (MM), **(B)** negative control BI-OPL in MM, **(C)** 943 (*Lachnellula* sp.) in R2A, **(D)** 943 (*Lachnellula* sp.) in MM, **(E)** 1205 (*Pseudogymnoascus pannorum*) in MM+gel, **(F)** 1205 (*Pseudogymnoascus pannorum*) in MM, **(G)** 737 (*Thelebolus globosus*) in MM, **(H)** 737 (*Thelebolus globosus*) in R2A, **(I)** 800 (*Neodevriesia* sp.) in MM, and **(J)** 800 (*Neodevriesia* sp.) in MM+gel.


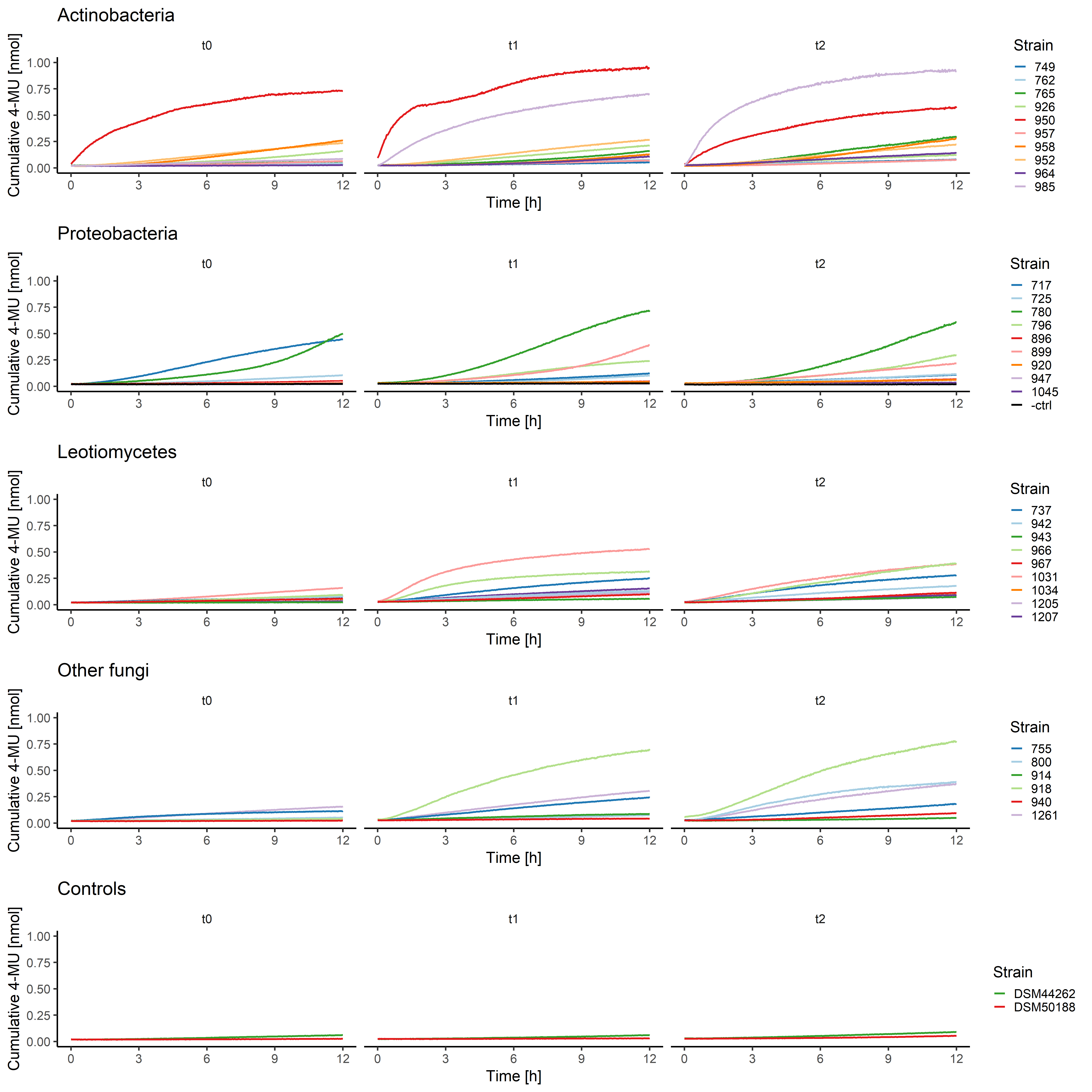


**Figure S9:** Cumulative 4-MU released by co-hydrolysis of 4-MUL and the polybutylene adipate-co-terephthalate (PBAT) matrix over time by microbial strains. The strains were incubated with ecovio^®^ in mineral medium (MM). Subsamples were taken at different timepoints and added to the polymer-coated 96-well plates. The increase in fluorescence intensity was measured for 12 h and converted into cumulative 4-MU released with a calibration curve. t0: directly after incubation; t1: 7 days after incubation; t2: 25 days after incubation. Only the means of all three replicates per strain are shown for ease of visual presentation. Note that for strain 920 (*Variovorax ginsengisoli*) only two replicates were used for timepoint 2 because one replicate was contaminated. Information about the microbial strains is given in Table 1.


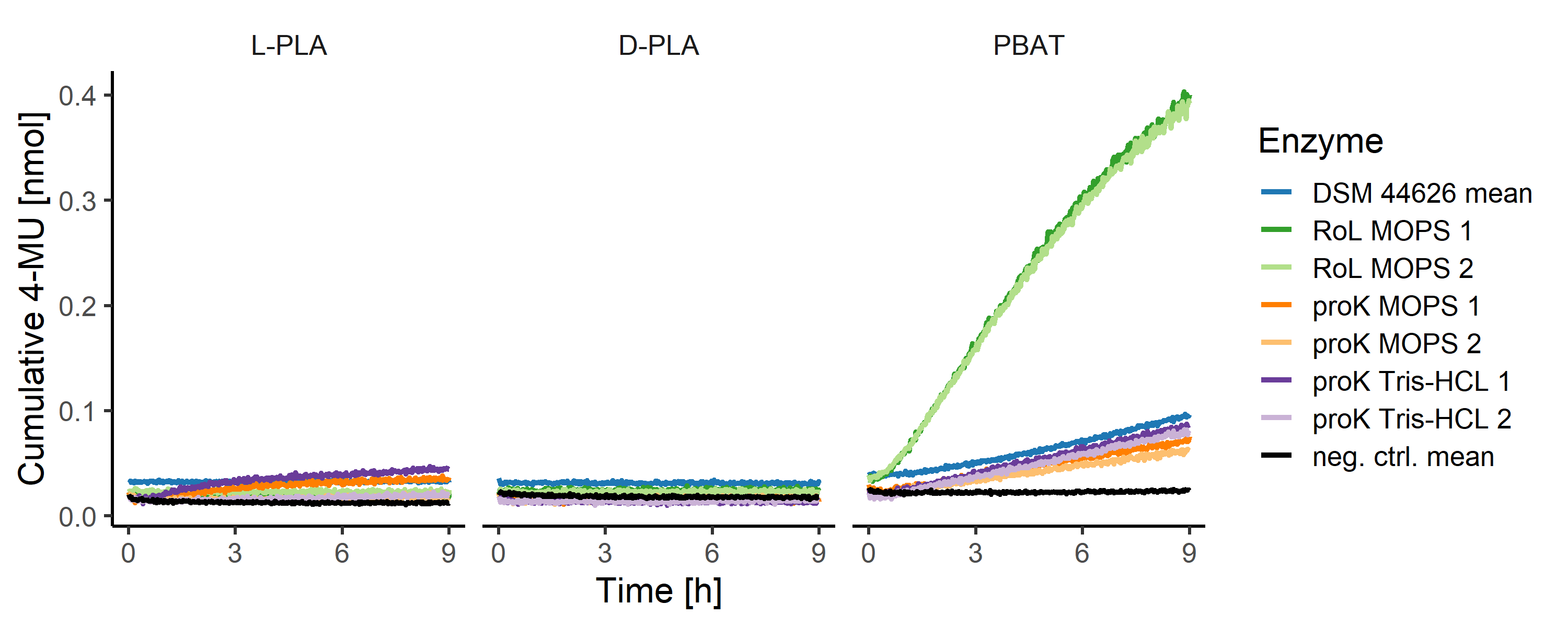


**Figure S10:** Cumulative 4-MU released by co-hydrolysis of 4-MUL and the (left) L-polylactic acid (L-PLA), (middle) D-polylactic acid (D-PLA), and (right) PBAT matrices over time by enzyme and microbial strain controls. Negative controls containing only culturing medium / buffer and plastic were processed in triplicate for mineral medium (MM), MOPS buffer and Tris-HCL buffer (only the mean of the MOPS buffer controls is shown). Enzyme assays were run in duplicates (numbers 1 & 2 in legend). The enzymes were added to the polymer-coated 96-well plates in a concentration of 12 µM. The assays with proK and RoL were run at 37°C and 30°C, respectively. Assays with proK were run in both MOPS (pH 7) and Tris-HCL (pH 8) buffers. Assays with PLA positive control strain DSM 44262 (*Amycolatopsis alba*) were run in MM at 30°C and the mean of three replicates is shown. The different panels show assays with 96-well plates coated with the different polymers. The increase in fluorescence intensity was measured for 9 h and converted into cumulative 4-MU released with a calibration curve. proK = *Tritirachium album* proteinase K; RoL = *Rhizopus oryzae* lipase.
