# Supplementary material for: Discovery of plastic-degrading microbial strains isolated from the alpine and Arctic terrestrial plastisphere
Source: Front Microbiol. 2023 May 10;14:1178474. doi: 10.3389/fmicb.2023.1178474 (PMC10206078; doi:10.3389/fmicb.2023.1178474)
Supplement: Supplementary file 2 [file data_sheet_2.docx]

**Supplementary tables**

| **Plastic** | **Polymer type** | **Product** | **Biodegradability** | **Form** | **Manufacturer** |
| --- | --- | --- | --- | --- | --- |
|  |  |  |  |  |  |
| Impranil^®^ DLN-SD | Polyester-polyurethane | commercial | in water (OECD 301) | dispersion | CSC Jäklechemie GmbH & Co. KG. |
| ecovio^®^ | PBAT + PLA | commercial | compostable  (EN 13432) | compostable waste bag | Petroplast Vinora AG |
| BI-OPL | PBAT + PLA | commercial | compostable (EN 13432) and in soil (EN 17033) | mulch film | Oerlemans Plastics BV |
| PE | LDPE | commercial | non-degradable | waste bag | TopPac AG |
| PBAT | PBAT (adipate: terephthalate 1:1) | pure | NA | pellets | BASF SE |
| L-PLA | 100% L-isomer PLA | pure | NA | pellets | Sulzer Ltd. |
| D-PLA | 100% D-isomer PLA | pure | NA | pellets | Sulzer Ltd. |
| L/D-PLA | 95% L-, 5% D-isomer PLA | pure | NA | pellets | Sulzer Ltd. |

**Table S1:** Plastic types used in the study.

Commercial products likely contain additives and the exact composition is not known, whereas pure means pure polymer without any additives. PE = polyethylene; LDPE = low-density polyethylene; PBAT = polybutylene adipate-co-terephthalate; PLA = polylactic acid; NA: not analyzed (biodegradable polymers, but no certificates are available because the materials are not commercial products).

**Table S2:** Differences in the weight loss of plastic films between the tested microbial strains and negative controls.

| **Strain** | ***P*** | | |
| --- | --- | --- | --- |
|  | **ecovio^®^** | **BI-OPL** | **PE** |
|  |  |  |  |
| 717 | 0.98 | 0.96 | 1.00 |
| 725 | 0.99 | 1.00 | 1.00 |
| 737 | 0.10 | **7.45×10^-05^** | 1.00 |
| 749 | 0.98 | 0.96 | 1.00 |
| 755 | 0.83 | 0.63 | 1.00 |
| 762 | **4.97×10^-03^** | 0.76 | 1.00 |
| 765 | 0.92 | 0.71 | 1.00 |
| 780 | 0.89 | 0.77 | 1.00 |
| 796 | 0.99 | 0.96 | 1.00 |
| 800 | **0.03** | **2.18×10^-19^** | 1.00 |
| 896 | 1.00 | 1.00 | 1.00 |
| 899 | 0.98 | 0.75 | 1.00 |
| 914 | 0.95 | 0.59 | 1.00 |
| 918 | **1.87×10^-03^** | 0.37 | 1.00 |
| 920 | 0.98 | 0.91 | 1.00 |
| 926 | 0.48 | 0.85 | 1.00 |
| 940 | 0.85 | 0.60 | 1.00 |
| 942 | **2.00×10^-04^** | 0.28 | 0.83 |
| 943 | **2.67×10^-08^** | **1.88×10^-35^** | 1.00 |
| 947 | 0.95 | 0.82 | 1.00 |
| 950 | 0.82 | 0.84 | 1.00 |
| 952 | 0.50 | 0.77 | 1.00 |
| 957 | 0.99 | 0.96 | 1.00 |
| 958 | 0.98 | 0.97 | 1.00 |
| 964 | **1.50×10^-07^** | 0.90 | 1.00 |
| 966 | **1.78×10^-07^** | 0.23 | 1.00 |
| 967 | **5.06×10^-06^** | 0.61 | 1.00 |
| 985 | 0.63 | **1.87×10^-05^** | 1.00 |
| 1031 | **4.85E×10^-07^** | 0.09 | 1.00 |
| 1034 | **3.11×10^-04^** | 0.19 | 1.00 |
| 1045 | 0.98 | 0.96 | 1.00 |
| 1205 | **9.98×10^-14^** | 0.07 | 1.00 |
| 1207 | 0.87 | **4.13×10^-03^** | 1.00 |
| 1261 | **3.00×10^-04^** | 0.60 | 1.00 |

*P*-values (*P* < 0.05 in bold) of pairwise t-tests, determined for each plastic, are given. Only the comparison of each strain with the negative controls is shown.

**Table S3:** Differences in the mass loss of individual plastic film components between selected microbial strains and negative controls.

| **A)** | |  | | | |
| --- | --- | --- | --- | --- | --- |
| **Main test** | |  | | | |
|  | | **PBAT** | | **PLA** | |
|  | | ***F*** | ***P*** | ***F*** | ***P*** |
| **Section 3.3** | **ecovio^®^** | 19.78 | **4.49*10^-10^** | 5.54 | **1.31*10^-4^** |
|  | **BI-OPL** | 161.50 | **1.42*10^-10^** | 26.48 | **8.75*10^-6^** |
| **Section 3.4** | **ecovio^®^** | 10.15 | **7.41*10^-5^** | 15.56 | **4.75*10^-6^** |
|  | **BI-OPL** | 10.59 | **4.51*10^-4^** | 13.85 | **1.24*10^-4^** |
|  | | | | | |
| **B)** | | | | | |
| **Pairwise tests** | |  | | | |
|  | | ***P*** | | | |
|  | | **ecovio^®^** | | **BI-OPL** | |
| **Strain vs. negative ctrl.** | | **PBAT** | **PLA** | **PBAT** | **PLA** |
| **Section 3.3** | |  |  |  |  |
| 918 *P. stoloniferum* | | 0.17 | 0.66 |  |  |
| 1261 *V. leptobactrum* | | 0.47 | 0.89 |  |  |
| 943 *Lachnellula* sp. | | **1.97*10^-7^** | **3.48*10^-3^** | **2.40*10^-10^** | **1.70*10^-5^** |
| 942 *P. pannorum* | | 0.10 | 0.55 |  |  |
| 966 *P. verrucosus* | | **4.02*10^-6^** | 0.38 |  |  |
| 967 *P. roseus* | | 0.43 | 0.86 |  |  |
| 1031 *P. verrucosus* | | **1.56*10^-7^** | **1.61*10^-3^** |  |  |
| 1034 *P. pannorum* | | **2.80*10^-5^** | 0.37 |  |  |
| 1205 *P. pannorum* | | **4.25*10^-4^** | 0.38 |  |  |
| 1207 *O. echinulatum* | |  |  | 0.56 | 0.93 |
| 800 *Neodevriesia sp.* | | **1.03*10^-4^** | 0.68 | **1.18*10^-7^** | **0.03** |
| 737 *T. globosus* | |  |  | **5.68*10^-3^** | 0.86 |
| 762 *U. tangerina* | | 0.69 | 0.52 |  |  |
| 985 *Amycolatopsis* sp. | |  |  | **2.53*10^-3^** | **2.10*10^-4^** |
| 964 *Streptomyces* sp. | | 0.78 | 0.80 |  |  |
| **Section 3.4** | |  |  |  |  |
| 943 *Lachnellula* sp. in MM | | **3.98*10^-4^** | **0.05** | 0.09 | 0.72 |
| 1205 *P. pannorum* in MM | | **3.98*10^-4^** | **3.88*10^-3^** |  |  |
| 964  *Streptomyces* sp. in MM | | 0.13 | 0.31 |  |  |
| 1205 *P. pannorum* in R2A | | 0.07 | 0.70 |  |  |
| 737 *T. globosus* in R2A | |  |  | **8.42*10^-5^** | **2.77*10^-5^** |
| 762 *U. tangerina* in R2A | | 0.93 | **8.81*10^-6^** |  |  |
| 964 *Streptomyces* sp. in R2A | | 0.07 | 0.48 |  |  |
| 800 *Neodevriesia* sp. in MM+gel | |  |  | **0.02** | 0.93 |

**(A)** The effect of the strain on the mass loss of the polymer components (PBAT and PLA) for ecovio^®^ and BI-OPL films in sections 3.3 and 3.4 was tested by ANOVA. F- and P-values are given (P < 0.05 in bold). **(B)** Pairwise comparisons of strains with the corresponding negative controls were done by pairwise t-tests. P-values (P < 0.05 in bold) are shown. Only films significantly reduced in the total weight from sections 3.3 and 3.4 were included in the analysis of the mass loss of individual film components.

| **A) Main test** |  | | | | | |  | | | | | |  |
| --- | --- | --- | --- | --- | --- | --- | --- | --- | --- | --- | --- | --- | --- |
|  | **ecovio^®^** | | | | | | **BI-OPL** | | | | | |  |
|  | ***F*** | | | ***P*** | | | ***F*** | | | ***P*** | | |  |
| **Strain** | 488.7 | | | **< 2.2×10^-16^** | | | 12.5 | | | **1.1×10^-6^** | | |  |
| **Medium** | 18 | | | **1.5×10^-7^** | | | 9.3 | | | **8.6×10^-5^** | | |  |
| **Strain × Medium** | 45 | | | **< 2.2×10^-16^** | | | 14.5 | | | **5.7×10^-11^** | | |  |
|  | | | | | | | | | | | | |  |
| **Pairwise tests** | | | | | | | | | | | | |  |
|  | ***P*** | | | | | | | | | | | |  |
| **B) Strain vs. negative ctrl.** | **MM** | | | **MM+gel** | | | **R2A** | | | **R2A+gel** | | |  |
| **ecovio^®^** |  | | |  | | |  | | |  | | |  |
| 1205 | **1.2×10^-08^** | | | 0.31 | | | **1.0×10^-04^** | | | 0.16 | | |  |
| 943 | **1.6×10^-06^** | | | 0.32 | | | 0.58 | | | 0.28 | | |  |
| 762 | 0.28 | | | 0.53 | | | **1.6×10^-04^** | | | 0.40 | | |  |
| 964 | **0.01** | | | 0.35 | | | **6.8×10^-04^** | | | 0.05 | | |  |
| **BI-OPL** |  | | |  | | |  | | |  | | |  |
| 943 | **0.02** | | | 0.24 | | | 0.95 | | | 0.97 | | |  |
| 800 | 0.93 | | | **4.5×10^-03^** | | | 0.96 | | | 0.23 | | |  |
| 737 | 0.95 | | | 0.94 | | | **1.96×10^-12^** | | | 0.91 | | |  |
| 985 | 0.89 | | | 0.99 | | | 0.44 | | | 0.99 | | |  |
|  |  |  |  | |  |  |  |  |  | |  |  | |
|  | ***P*** | | | | | | | | | | | | |
| **C) Media** | **negative ctrl.** | **1205** | **943** | | **762** | **964** | **negative ctrl.** | **943** | **800** | | **737** | **985** | |
| MM vs. MM+gel | 0.95 | **1.47×10^-07^** | **7.49×10^-05^** | | 0.76 | 0.20 | 0.99 | 0.51 | **0.02** | | 0.99 | 0.95 | |
| MM vs R2A | 0.83 | **4.08×10^-03^** | **3.61×10^-05^** | | **2.51×10^-03^** | 0.23 | 0.99 | 0.07 | 0.99 | | **2.84×10^-12^** | 0.82 | |
| MM vs R2A+gel | 0.69 | **1.97×10^-06^** | **3.32×10^-04^** | | 0.84 | 0.97 | 0.99 | **0.05** | 0.47 | | 0.98 | 0.95 | |
| MM+gel vs. R2A | 0.87 | **1.86×10^-03^** | 0.84 | | **8.26×10^-04^** | **0.01** | 0.99 | 0.49 | **0.02** | | **3.50×10^-12^** | 0.45 | |
| MM+gel vs. R2A+gel | 0.75 | 0.48 | 0.69 | | 0.59 | 0.22 | 1.00 | 0.41 | 0.24 | | 0.99 | 0.99 | |
| R2A vs. R2A+gel | 0.87 | **0.02** | 0.52 | | **0.01** | 0.22 | 0.99 | 0.99 | 0.45 | | **4.07×10^-12^** | 0.47 | |

**Table S4:** Differences in the weight loss of plastic films between selected microbial strains and negative controls cultured in different media.

**(A)** Effects of strain, medium and the interaction between strain and medium were tested by ANOVA. *F*- and *P*-values are given, with *P* < 0.05 in bold. **(B)** Pairwise comparisons of selected strains with the negative controls in the corresponding medium were done by pairwise t-tests. *P*-values (*P* < 0.05 in bold) are shown. (C) The impact of the culturing medium was tested by comparing the tested media for each strain with pairwise t-tests. *P*-values (*P* < 0.05 in bold) are given.

**Table S5:** Differences in the weight loss of plastic films between microbial strains cultured in test tubes and Petri dishes.

| **Strain** |  |  |
| --- | --- | --- |
| **ecovio^®^** | **t** | ***P*** |
| negative controls | 0.85 | 0.78 |
| 1205 | -6.96 | **1.12×10^-03^** |
| 943 | -2.17 | **0.05** |
| 762 | 0.71 | 0.74 |
| 964 | 0.43 | 0.66 |
| **BI-OPL** |  |  |
| negative controls | 3.12 | 0.98 |
| 943 | 3.05 | 0.98 |
| 800 | 6.50 | 1.00 |
| 737 | 2.41 | 0.96 |
| 985 | 5.09 | 1.00 |

Differences in weight loss between culturing microbial strains in test tubes and Petri dishes were assessed by one-sided t-tests. t- and *P*-values (*P* < 0.05 in bold) are given. Negative t-values indicate a greater weight loss for culturing in Petri dishes.

**Table S6:** Annotated chemical structures of the internal standard, 1,4-dinitrobenzene (DNB), solvent and polymer components of ecovio^®^ and BI-OPL.

| 1,4-dinitrobenzene (DNB) |
| --- |
| 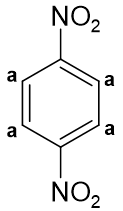 |
| CHCl_3_ |
| 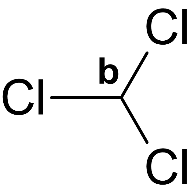 |
| PBAT (shifted) |
| 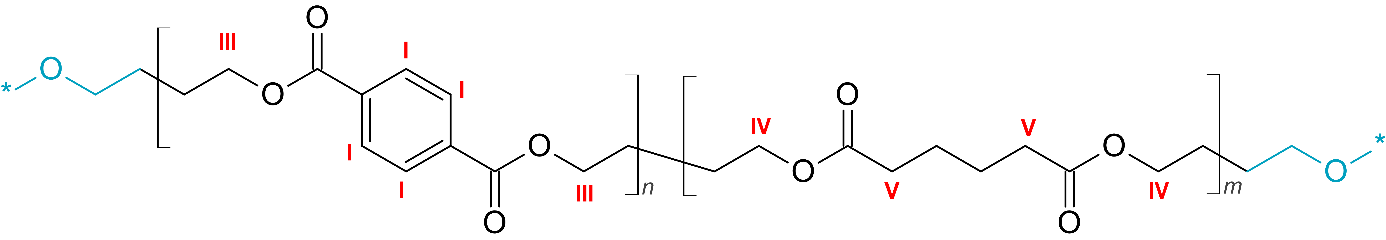 |
| PLA |
| 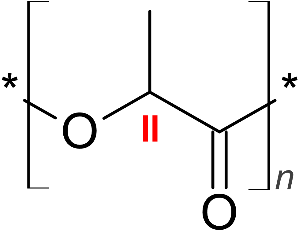 |

The structure of poly(butylene adipate-co-terephthalate) (PBAT) has been shifted to allow the unambiguous annotation of protons III and IV (see Figure S1). All figures were generated using ChemSketch v2021.2.1.
