# Supplementary material for: Discovery of plastic-degrading microbial strains isolated from the alpine and Arctic terrestrial plastisphere
Source: Front Microbiol. 2023 May 10;14:1178474. doi: 10.3389/fmicb.2023.1178474 (PMC10206078; doi:10.3389/fmicb.2023.1178474)
Supplement: Supplementary file 3 [file data_sheet_3.docx]

**Supplementary Materials and Methods: cultivation media used in this study**

Several different solid and liquid media were used for the isolation of microbial strains and plastic degradation assays. Information about isolation media for each strain is listed in Table 1. R2A agar (NutriSelect^®^ Plus, Merck KgaA, Darmstadt, Germany) was used for isolating most of the microbial strains. Per liter it contained 15 g agar, 0.5 g casein acid hydrolysate, 0.5 g dextrose, 0.3 g dipotassium phosphate, 0.024 g magnesium sulfate, 0.5 g proteose peptone, 0.3 g sodium pyruvate, 0.5 g soluble starch, and 0.5 g yeast extract. For isolating strain 780, a 1:10 diluted R2A agar was used, and agar (Merck KgaA, Darmstadt, Germany) was added to reach a final concentration of 15 g/l. For the isolation of strain 940, malt extract agar (MEA; Carl Roth GmbH & Co. KG, Karlsruhe, Germany) was used. Per liter it contained: 12.75 g malt extract, 0.78 g gelatin peptone, 2.35 g glycerol, 2.75 g dextrin, and 15 g agar. For the isolation of strain 1205, a soil extract agar with lactic acid added to it was used (SELAA). For this, an aqueous soil extract was prepared by shaking 80 g of active-layer soil from Villum, Greenland (Adamczyk et al., 2020) in 500 ml of deionized H_2_O for 30 min on a rotary shaker. Agar (15 g/l; Merck KgaA, Darmstadt, Germany) and DL-lactic acid (0.5 ml/l, 90%; Merck KgaA, Darmstadt, Germany) were added to the soil extract before autoclaving. The pH was adjusted to 7 with NaOH. A mineral medium (MM) without a carbon source, adapted from Brunner et al. (2018), was prepared for the initial plastic degradation screening. Per liter it contained: 3 g NH_4_NO_3_, 5 g K_2_HPO_4_^-^, 1 g NaCl, 0.2 g MgSO_4_·7H_2_O, 1 ml FeCl_3_ (1.2%), 1.546 mg H_3_BO_3_, 0.845 mg MnSO_4_·H_2_O, 0.575 mg ZnSO_4_·7H_2_O, 0.125 mg CuSO_4_·5H_2_O, and 0.018 mg (NH_4_)_6_Mo_7_O_24_·4H_2_O. For the polyurethane degradation screening, 15 g/l agar was added before autoclaving, and 10 ml/l Impranil^®^ was added to the MM after autoclaving (= MM+Imp). To determine the effect of the culturing medium on the degradation of plastics, MM and R2A broths (Lab M Limited, Lancashire, UK) were used. In addition, 0.3% gelatin (Migros, Zurich, Switzerland) was added to the MM and R2A broths as a possible inducer of secreted enzymes (= MM+gel and R2A+gel).
